# A solar panel-origin microalga, *Coelastrella thermophila* D14, with high potential for wastewater biotechnology

**DOI:** 10.1007/s00253-025-13618-8

**Published:** 2025-11-24

**Authors:** Sara Baldanta, Alice Ferreira, Arantxa Marco Vinuesa, Isabel García García, Luisa Gouveia, Juana María Navarro Llorens, Govinda Guevara

**Affiliations:** 1 Department of Biochemistry and Molecular Biology, Complutense University of Madrid, Madrid, 28040 Spain; 2 Department of Genetics, Physiology and Microbiology, Complutense University of Madrid, Madrid, 28040 Spain; 3 Bioenergy and Biorefineries Unit, National Laboratory of Energy and Geology, Estrada Do Paço Do Lumiar 22, Lisbon, 1649-038 Portugal; 4 GreenCoLab - Green Ocean Technologies and Products Collaborative Laboratory, CCMAR, University of Algarve, Campus de Gambelas, Faro, 8005-139 Portugal; 5 Institute for Integrative Systems Biology (I2SysBio, CSIC-UV), Consejo Superior de Investigaciones Científicas, Valencia, 46980 Spain

**Keywords:** Xero-tolerant microalga, Solar panel isolation, *Coelastrella thermophila* D14, Piggery wastewater, Biostimulant, Genetic transformation

## Abstract

**Abstract:**

Extremophilic environments are rich reservoirs for discovering microorganisms with vast biotechnological potential. Among these, microalgae stand out for their pivotal role in sustainable wastewater treatment and nutrient recycling. This study introduces *Coelastrella thermophile* D14, a microalga isolated from a solar panel, identified through morphological studies and genomic sequencing. The genus *Coelastrella* has been characterized and classified as highly productive strains valuable for biofuel and bioproduct generation as well as for their ability to produce significant amounts of carotenoids. Experiments revealed the extraordinary resilience of this strain to prolonged desiccation and high-strength piggery wastewater. Notably, D14 cultivated in 10% pig effluent exhibited biostimulant properties, achieving a germination index 23% higher than the control on *Lepidium sativum*. In a groundbreaking development, we have successfully established an *Agrobacterium*-mediated transformation protocol for *C. thermophila* D14, optimizing key parameters for effective T-DNA transfer. This marks a pioneering achievement within the genus *Coelastrella*. These findings highlight the significant potential of D14 as a robust platform for future biotechnological applications, opening new opportunities for innovative solutions, especially in environmental protection and sustainable agriculture.

**Graphical Abstract:**

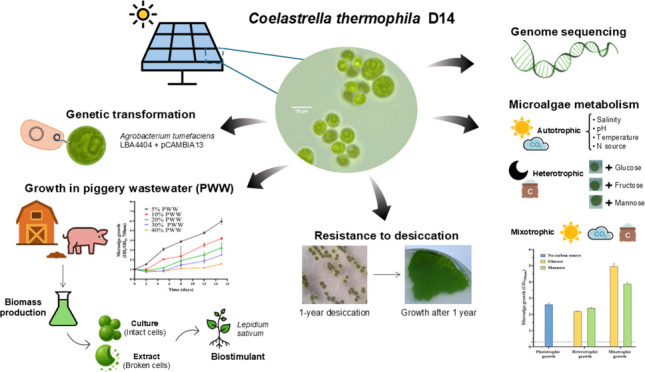

**Key points:**

• *First microalga from solar panel biofilm: Coelastrella sp. D14 isolated and characterized.*

• *Strain D14 tolerates prolonged desiccation and grows well in piggery wastewater.*

• *Stable Agrobacterium-mediated transformation enables future metabolic engineering.*

**Supplementary information:**

The online version contains supplementary material available at 10.1007/s00253-025-13618-8.

## Introduction

Water scarcity and pollution, recognized as significant environmental concerns, have garnered widespread attention, prompting efforts towards finding solutions (Hussain et al. 2021). Microalgae are one of the most attractive biological agents to address the water pollution problems and greenhouse effect (El-Sheekh et al. 2025; Ferreira et al. 2018, 2019; François et al. 2025; Li et al. 2024; Navarro-López et al. 2020; Zhao et al. 2025). Microalgae are a highly diversified group of photosynthetic microorganisms adapted to a wide range of ecological habitats that utilize solar energy to generate biomass. They stand out for their low nutritional needs, not depending on arable land nor potable water, the ability to grow under several stresses and the possibility of being harvested daily (Rizwan et al. 2018; Tang et al. 2020). Additionally, various microalgal species have demonstrated the capability to thrive in municipal or industrial wastewaters, effectively eliminating organic carbon, nitrogen, and phosphorus, while fixing CO_2_. Many industries use microalgal feedstocks to get high-value products from the biomass such as antioxidants, lipids, vitamins, pigments, or carbohydrates, as well as agricultural products and biofuels, to improve the overall economics (Dutta et al. 2025; Kaur et al. 2025; Lu et al. 2024; Nayana et al. 2022; Sudhakar et al. 2019).

Microalgal growth depends on both chemical and physical factors such as the type and concentration of carbon sources and minerals present in the medium, light intensity and regime (dark/light), pH, agitation, or temperature (Singh et al. 2015). For instance, a shortage in nitrogen or phosphorus alters the biochemical composition of the microalgae but also causes a drop in the growth rate (Procházková et al. 2014). Similarly, physical parameters affect the biomass production depending on the microbial species (Daneshvar et al. 2021; Elisabeth et al. 2021; Khanra et al. 2021). The ability of microalgae to acclimate to demanding wastewater conditions and endure oxidative stress particular to these environments differs among species. However, minimizing the cost of biomass production must be considered, and therefore, an equilibrium between growth and the use of low-cost media must be reached. This strategy allows both (i) wastewater remediation by recovering nutrients and removing pollutants from the environment and (ii) the use of the biomass produced for different applications such as biofertilizers, bioplastics, or cosmetics (Ferreira et al. 2018, 2017; García et al. 2018; Posadas et al. 2017; Viegas et al. 2021).

The use of biofertilizers and biostimulants is of paramount importance in mitigating the use of chemical agents that can potentially result in environmental contamination, ultimately affecting the quality of food production. Ongoing endeavors are focused on broadening the application of these natural biostimulants in agricultural practices (González-Pérez et al. 2021; Navarro-López et al. 2020; Sánchez-Quintero et al. 2023) under strict legislations and regulations that depends on the country (Su et al. 2023). The use of microalgae as biostimulants has acquired importance for their role in the sustainability and circular bioeconomy agenda (Ajeng et al. 2022; Sánchez-Quintero et al. 2023; Vangenechten et al. 2025). This is because they are capable of sequestering CO_2_, they can survive in challenging environments such as waste effluents and their easier cultivation compared to macroalgae (Sánchez-Quintero et al. 2023).

*Coelastrella* (Chlorophyta phylum, Sphaeropleales order, Scenedesmaceae family) is a genus of green microalgae with reported applications for bioremediation and value-added products such as UV-protective compounds among others (Zaytseva et al. 2021). *Coelastrella* is also a good renewable energy resource feedstock with a total of 18% of their biomass made up of lipids beneficial for biodiesel conversion (Nayana et al. 2022). This genus includes mainly unicellular, ellipsoidal cells with peculiar apical wart-like wall thickenings (Goecke et al. 2020; John 2002; Maltsev et al. 2021; Wang et al. 2019a). It can be often found in subaerial and terrestrial habitats, and it is universally distributed from the arctic boreal zone to tropical zones (Nayana et al. 2022).

The bioprospection of strains in extremophilic environments has discovered strains with high resistance to various stresses such as extreme dehydration, high salinity, and excessive light exposure. Some examples are as follows: *Coelastrella thermophila* var. globulina isolated from an Algerian hot spring producing n-6 and n-3 polyunsaturated fatty acid of commercial interest (Boutarfa et al. 2022); *Coelastrella terrestris* collected from red mucilage in a glacier foreland in Iceland for adonixanthin production (Doppler et al. 2022); a *Coelastrella* sp. isolated from an ammonia-rich environment for piggery wastewater treatment and biodiesel production (Lee et al. 2021). The study of their genomes could give hints of the genes involved in these processes apart from their biotechnological potential although, so far, only a few *Coelastrella* genomes have been fully sequenced (*Coelastrella* sp. MACC-549: GCA_018290735.1; *Coelastrella* sp. M60: GCA_001630525.1; *Coelastrella* sp. UTEX B 3026: GCA_002588565.1).

One of the intriguing extreme environments under investigation is that of solar panels, which endure various stresses including high irradiation, temperature fluctuations, and desiccation (Dorado-Morales et al. 2016; Porcar et al. 2018; Tanner et al. 2018). This study focuses on the identification and characterization of a *Coelastrella* strain isolated from a solar panel. The biotechnological potential of this novel strain was evaluated for wastewater treatment of piggery wastewater and biostimulant products. Moreover, it was the first time that the genetic transformation of this strain has been achieved. To date, despite studies demonstrating that the *Coelastrella* genus holds great promise in fields such as biofuel production, biomass generation, CO_2_ capture, and pigment production (Nayana et al. 2022; Neofotis et al. 2016), no research has explored its genetic manipulation, which begins with the ability to transform the strain using vectors of interest. This work presents, for the first time, a protocol for its genetic manipulation, opening a window of opportunity for targeted metabolic engineering and the development of novel biotechnological applications.

## Materials and methods

### Strain isolation and culture conditions

The microalga used in this study, *Coelastrella thermophila* D14, was isolated from a solar panel in Valencia (Spain), as previously described (Baldanta et al. 2023). *C. thermophila* D14 was grown in BG11 medium on 1.5% agar plates or liquid medium at 30 °C ± 2 °C under 100 μE m^−2^ s^−1^ of continuous white light, under orbital shaking (150 rpm). The BG11 medium contained 1.5 g L^−1^ NaNO_3_; 0.02 g L^−1^ Na_2_CO_3_; 0.03 g L^−1^ K_2_HPO_4_; 0.075 g L^−1^ MgSO_4_ * 7 H_2_O; 0.036 g L^−1^ CaCl_2_ * 2 H_2_O; 1 g L^−1^ Na_2_EDTA * 2 H_2_O; 1.81 g L^−1^ MnCl_2_* 4 H_2_O; 0.05 g L^−1^ CoCl_2_* 6 H_2_O; 0.039 g L^−1^ Na_2_MoO_4_*H_2_O; 0.08 g L^−1^ CuSO_4_* 5 H_2_O; 0.22 g L^−1^ ZnSO_4_ * 7 H_2_O; 2.86 g L^−1^ H_3_BO_3_; 6 g L^−1^ citric acid; and 6 g L^−1^ ferric ammonium citrate (PhytotechLabs) (Rippka et al. 1979). BG11 medium was buffered to pH 7.5 with 10 mM HEPES. The cultured algal cells were observed and photographed under a microscope (Leica, model DM750), to ensure no contamination with other microorganisms. Axenic strains were stored at − 80 °C in BG11 medium supplemented with 5% (v/v) DMSO. The strain *Coelastrella thermophila* D14 is available upon request from the corresponding author and will be provided for research purposes to any qualified investigator wishing to reproduce or build upon the experiments described in this study.

Cultures of *C. thermophila* D14 were harvested at different growth phases and preparations of these cultures were photographed and then processed by the LAS V4.2 software. ImageJ software was used to measure the cell size.

### Genus, species identification, and draft genome

Isolated D14 was previously identified using primers for 18rRNA amplification (18S-Fw 5′-GTCAGAGGTGAAATTCTTGGATTTA-3′, 18S-Rv 5′-AGGGCAGGGACGTAATCAACG-3′) (Baldanta et al. 2023).

Then, the genome sequence was performed by MicrobesNG (https://microbesng.com), obtaining 250-bp paired-end reads. Raw reads were trimmed with Trimmomatic v0.39 (Bolger et al. 2014) to remove low-quality sequences and adapter contamination. Clean reads were then assembled with SPAdes v3.13.0 (Bankevich et al. 2012) to create a de novo draft genome. RagTag v2.1.0 (Alonge et al. 2022) was used to improve the quality of the obtained assembly, using the *Coelastrella* sp. strain UTEX B 3026 draft genome (PRJNA401156 at NCBI) for homology-based scaffolding. AUGUSTUS v3.1 (Stanke and Morgenstern 2005) was used for gene prediction, using the “*Chlamydomonas*” database, and blast2go v6.0.3 (Conesa et al. 2005) was used for functional annotation of the identified genes, restricting the analysis to Viridiplantae sequences (*E*-value < 10^−5^; > 40% identity). Then, BBMap (BBTools) v39.06 (sourceforge.net/projects/bbmap/) was used to assess the draft genome’s quality and BUSCO v5.7.0 (Manni et al. 2021) was used to evaluate its completeness, using the “chlorophyta_odb10” lineage database. Finally, Blast2GO (Conesa et al. 2005) was used for the functional annotation step, and the web-based server KASS (Moriya et al. 2007) was used to identify the most represented KEGG pathways. The draft genome was deposited at https://zenodo.org/records/11631735 (10.5281/zenodo.11631734).

### Autotrophic growth

Axenic *C. thermophila* D14 was inoculated into 20 mL of BG11 medium in 100-mL Erlenmeyer flasks to an initial optical density at 750 nm (OD_750nm_) of 0.05 and the growth of the microalga was tested at different conditions, including salinity, pH, nitrogen sources, and temperature. Conditions were chosen according to other works on microalgae characterization (Church et al. 2017; Taton et al. 2012; Xia et al. 2014). To assess growth at different salt concentrations, BG11 medium was prepared containing 0.1, 0.25, 0.5, or 1 M of NaCl. We chose low concentrations, as the BG11 lacks NaCl, a concentration that is close to seawater, and a high concentration, used in other saline microorganisms (Church et al. 2017; Xia et al. 2014). The influence of pH on growth was explored in BG11 buffered to pH 4, 6.5, 9, and 11 with 10 mM Tris adjusted to each pH. During the growth of the microalgae, the pH was not controlled or adjusted to the initial condition. To examine the strains for growth on different nitrogen sources, BG11 was modified by replacing the 16 mM of NaNO_3_ with 16 mM of ammonium chloride or urea. Tolerance to urea was determined by adding this compound to final concentrations of 8 and 16 mM. These conditions were selected as they have been used in other photosynthetic microorganisms (Taton et al. 2012; Xia et al. 2014). For temperature experiment tests, 100-mL Erlenmeyer flasks with an initial OD_750nm_ of 0.20 were used. The temperature effect on growth was evaluated at 4, 30, 40, and 50 °C, keeping the other conditions constant. Cell growth was monitored by measuring OD_750nm_ for a 10-day period in a Beckman Coulter Du800 spectrophotometer. In all the growth experiments, three biological replicates were performed. As a control for all experiments, strains grown under routine conditions (BG11, pH 7.5, 30 °C, 150 rpm, and continuous light 100 μE m^−2^ s^−1^) were used.

A relationship between cell densities and OD_750nm_ values was defined, using a hemocytometer for cell counting. Growth rate (*r*) was determined by plotting the log OD versus time and calculating the slope in the linear portion, related to the exponential growth. The beginning of the growth phase was considered when the growth of the microalga was appreciable. Doubling time corresponds to log2/*r*. Finally, the biomass dry weight and the ash free dry weight (AFDW) were determined gravimetrically by drying the samples at 105 °C overnight and incinerating at 550 °C for 1 h, respectively.

### Heterotrophic and mixotrophic growth

First, to assess the heterotrophic growth of *C. thermophila* D14, BG11 agar plates were prepared at final concentrations of 10 mM with different carbon sources: glucose, sucrose, lactose, arabinose, maltose, fructose, galactose, mannose, and glycerol. The tests were performed with spots of 10 μL at OD_750nm_ = 1 onto BG11 plates to reduce the possibility of contamination. Plates were incubated at 30 °C in darkness for 30 days. Furthermore, the photosynthesis inhibitor DCMU (3-(3,4-dichlorophenyl)−1,1-dimethylurea) was added at 10 μM guarantee exclusively heterotrophic growth. The cell growth was evaluated by checking the appearance of colonies after the incubation period.

Once the sugars were selected for *C. thermophila* D14 cultivation, the heterotrophic and mixotrophic growth in liquid medium was evaluated. Axenic *C. thermophila* D14 was inoculated into 20 mL of BG11 medium in 100-mL Erlenmeyer flasks to an initial OD_750nm_ of 0.3 and grown in light conditions with no sugar, and glucose or mannose at 10 mM (mixotrophic growth). In parallel, the same conditions were used adding the photosynthesis inhibitor DCMU at 10 μM (heterotrophic growth). Cell growth was monitored by measuring OD_750nm_ for a 7-day period. In all the growth experiments, three biological replicates were performed. Cultures were examined under a microscope, and microalgae were plated on LB to ensure they were free of contaminant bacteria before the experiments.

### Desiccation-tolerance test

To assess the desiccation tolerance of *C. thermophila* D14, the survival after several months dry was tested in agar plates, as described previously (Katoh et al. 2003). *C. thermophila* D14 was grown on 9–10-mL of BG11 agar plates (6-cm diameter) under continuous light (60–80 μE m^−2^ s^−1^) at 30 °C for 2 weeks at 30–35% relative humidity. Then, plates were left to be air-dried under routine growth conditions by removing the parafilm from the Petri dishes. After about 15 days, dried cultures were stored in the laboratory bench at room temperature for 3 and 7 months, and 1 year. For the 1-year dried samples, some samples were maintained in parallel under routine growth conditions. For rehydration, the dried samples were soaked with 1 mL of sterile water for 15 min at room light, streaked on BG11 plates, and incubated under the same initial conditions (60–80 μE m^−2^ s^−1^ and 30 °C). Results were observed after 2–3 weeks. We chose the freshwater cyanobacterium *Synechocystis* sp. PCC 6803 as a negative control because it has been previously reported and validated to lack prolonged resistance to desiccation tolerance, following the same approach used in this work (Baldanta et al. 2023; Katoh et al. 2003).

## Wastewater characterization

The piggery wastewater (PWW) was collected from a stabilization pond in a local pig farm from Valorgado in Herdade do Pessegueiro (39°00009.000 N, 8°38,045.500 W, Glória do Ribatejo, Portugal). This effluent corresponds to the liquid fraction of pig slurry after separation (sieve 1–10 mm) from solid manure. The nutrient composition of PWW was determined by standard methods. The Kjeldahl nitrogen (TKN) was determined by a modified Kjeldahl method adapted from the standard method 4500-Norg B (Clesceri et al. 1988). Ammonium nitrogen was quantified by titration after a distillation step based on standard methods 4500-NH 3 B and C (Clesceri et al. 1988). A commercial kit was used for the measurement of phosphorus (Phosver 3-Powder Pillows, Cat. 2125–99, HACH) using a HACH DR/2010 spectrophotometer, at 890 nm. Chemical oxygen demand (COD) determination was carried out according to the open reflux method—Method 5220-B. The effluent composition was as follows: 1333 ± 6 mg N L^−1^(TKN); 1281 ± 1.4 mg NH_4_^+^ L^−1^; 218 ± 5 mg PO_4_^3−^ L^−1^; and 4396 ± 94 mg O_2_ L^−1^ (COD), with a pH of 7.72.

### Assessment of microalgae growth potential in wastewater

A *screening* was carried out to determine if *C. thermophila* D14 was able to grow in PWW. Different dilutions (5, 10, 20, 30, and 40%) of PWW were prepared with tap water. The microalga was inoculated at OD_750nm_ of 0.2 in 20 mL of prepared diluted media in 50-mL Erlenmeyer flasks, in duplicate. The cultures were kept at room temperature (23–25 °C), under a constant light intensity of 41 μE m^−2^ s^−1^, and orbital agitation at 150 rpm (New Brunswick Scientific Co, USA). Cell growth was monitored by measuring OD_750nm_ and pH for a 15-day period.

## Biomass production

To obtain biomass, the microalga cultures were initially cultivated in 1-L bubble-column photobioreactors (PBRs) using 1:20 (PWW) or BG11 as medium. The cultures were maintained at room temperature (23–25 °C) under continuous illumination at an average light intensity of 60 μE m^−2^ s^−1^. The aeration was supplied at 0.15 vvm (air volume (L) per volume of culture medium (L) per minute (m)) by aquarium pumps.

Then, microalga cultures were transferred to 4-L bubble columns photobioreactors (PBRs) using 5% and 10% PWW as media, at a working volume of 1 L. The cultures were maintained in the same conditions previously described. After 9 days, the biomass was collected by centrifugation (Sigma 6-16KS, 10,000 × g, 10 min).

### Germination index

The biostimulant activity of the microalga *C. thermophila* D14 was determined by measuring the germination index of garden cress (*Lepidium sativum*) seeds, according to the method described by Zucconi et al. (1981).

Microalga culture (whole biomass) and broken-cells obtained from the growth at different conditions (BG11, 5%, 10%, and 20% PWW) were tested at different concentrations (0.1, 0.5, 1, and 2 g L^−1^). Broken-microalga samples were prepared by submitting the harvested biomass to high-pressure homogenization at 1200 bar for 1 cycle (Ferreira et al. 2022). Microalga suspensions were prepared in distilled water to the desired concentrations. A total of 32 treatments were tested (Fig. S1, Supplementary Material A).

The germination experiments were carried out in sterilized rectangular Petri dishes (10 mm × 17 mm) with Whatman No. 5 filter papers wetted with 7 mL of each treatment solution, with 10 seeds per dish in duplicates. Distilled water was used as the negative control. All samples were incubated in the growing chamber (FITOCLIMA S600 PL) at 25 °C in the dark for 3 days and the Petri dishes were kept in a vertical position. At the end of 3 days, the seedlings were photographed and measured with the program ImageJ (Rasband 1997). Results were registered for comparison between the microalga treatments and the control with distilled water.

Finally, the germination index was determined by Eq. (1), where *G* and *L* are the number of germinated seeds and their length in the case of the microalgal cultures and *G*_*w*_ and *L*_*w*_, in the control (distilled water), respectively (Zucconi et al. 1981). The data shown in the germination index experiments is, therefore, the result of the measurement of 20 seeds for each treatment.

1 $$GI \left(\%\right)=\frac{G \times L}{{G}_{w} \times {L}_{w}}\times 100$$

## Genetic transformation

*Agrobacterium tumefaciens* strain LBA4404, which has been reported to be the ideal strain for the transformation of other microalgae in previous studies (Cheng et al. 2012; Rajam and Kumar 2006), was used in this work, as there are no other works for *Coelatrella* genus transformation. *Agrobacterium* cultures and plates were grown at 30 °C in Luria broth (LB). Binary vector pCAMBIA1301 was used for the study (CAMBIA, Australia), which harbors *hptII* (hygromycin phosphotransferase) as antibiotic marker gene and b-glucuronidase (GUS) as reporter gene driven by CaMV 35S promoter (GenBank accession: AF234297.1). The binary vector was electroporated into *A. tumefaciens* strain LBA4404 using the MicropulserTM electroporator (Biorad, Hercules, USA) using 2.5 kV, 200 oms, and 50 µF. Transformants were selected with 50 mg L^−1^ rifampicin and 50 mg L^−1^ kanamycin and then maintained at − 80 °C in 20% (v/v) glycerol.

An antibiotic sensitivity test was performed prior to the transformation process. The sensitivity of *A. tumefaciens* to the antibiotic cefotaxime was evaluated by adding 200 µL of *Agrobacterium* culture (OD_600nm_ = 1.0) to 5 mL of LB supplemented with varying concentrations of cefotaxime (0, 100, 200, and 500 mg L^−1^). The growth of *Agrobacterium* at each concentration was then measured spectrophotometrically at OD_600nm_ after 2 days.

To assess the impact of cefotaxime on the viability of *C. thermophila* D14, a microalga culture was plated on solid BG11 with cefotaxime 500 mg L^−1^. For determining the minimum inhibitory concentration of hygromycin B, *C. thermophila* D14 cells were plated on solid BG11 medium supplemented with 500 mg L^−1^ cefotaxime and various concentrations of hygromycin B (10, 20, 50 mg L^−1^). Each treatment was tested in triplicate. After incubating the agar plates for 2 days in the dark at 30 °C, they were exposed to light for 15 days.

Based on another microalga transformation protocol (Cha et al. 2012), several factors were examined: bacterial density, duration of pre-culture, co-cultivation period, pH of the co-cultivation medium, and acetosyringone concentration. To investigate the impact of each factor on transformation frequency, one factor was altered while keeping other factors constant (Fig. S2, Supplementary Material A), as determined by preliminary testing results.

Single colonies of *Agrobacterium tumefaciens* LB4404 strain harboring the vector pCAMBIA1301 were utilized to inoculate 10 mL of LB supplemented with 5 mM glucose, 50 mg L^−1^ rifampicin, 50 mg L^−1^ streptomycin, and 50 mg L^−1^ kanamycin. The inoculated broth was grown overnight in a rotary shaker at 30 °C with agitation at 200 rpm in darkness. Five milliliters of this overnight culture was employed to inoculate 50 mL of the same medium, which was then incubated in the dark at 27 °C with shaking at 200 rpm until OD_600nm_ = 0.8–1.2. The bacterial culture was harvested by centrifugation, washed once with induction medium (BG11 plus the desired acetosyringone concentration), and diluted to the desired OD_600nm_ (different concentrations of *A. tumefaciens*) with induction medium.

Before co-cultivation, 5 × 10^6^ *C. thermophila* D14 cells from a logarithmic growth phase culture (OD_750nm_ = 0.5–1.0) in BG11 were cultured for 5 days on BG11 solidified with 1.2% (w/v) bacto-agar at 30 °C. On the day of co-cultivation, the algal cell pellet was collected with induction medium and 200 µL of bacterial suspension was added. The co-cultures were plated in induction medium solidified with 1.2% (w/v) bacto-agar and incubated for 3 or 5 days at 25 °C in darkness. Afterwards, the cells were collected using BG11 supplemented with 500 mg L^−1^ cefotaxime in a total volume of 7 mL. They were incubated again in darkness at 25 °C for 2 days to eradicate *Agrobacterium*. Finally, the culture was plated on selective media containing 20 mg L^−1^ hygromycin B and 500 mg L^−1^ cefotaxime and incubated at 25 °C in the dark for 2 days before exposure to light for 15 days. Resistant colonies were propagated on selective media, grown in liquid media with antibiotics and utilized for PCR analysis. Detection of contaminating *Agrobacterium* was performed by growing cells on LB agar plates for at least 7 days at 25 °C in the dark.

### PCR analysis of transformants

PCR analysis was carried out in a 50 µL reaction containing 20 μL DNA, 0.5 mM of each primer, and 1X NZYTaq II 2 × Green Master Mix (NZYTECH). Two fragments present in the plasmid were amplified. The primers used to amplify a 327-bp fragment of the *lacZ* gene a marker for cloning were CH283 (5′-GCGCGTTGGCCGATTCATTA-3′) and CH179 (5′-GCA AGG CGA TTA AGT TGG GTA ACG C-3′) while the 149-bp fragment was amplified using F24 (5′-CGC CAG GGT TTT CCC AGT CAC GAC-3′) and R24 (5′-AGC GGA TAA CAA TTT CAC ACA GGA-3′). PCR conditions were initial denaturation at 94 °C for 10 min, 30 cycles of 94 °C for 30 s, 56 °C for 30 s, and 72 for 30 s (annealing and extension) followed by a final cycle at 72 °C for 7 min. Amplified products were separated on 2% agarose gel in 1X TAE buffer with NZYTECH II ladder.

## Statistical analysis

Statistical analysis of data from *C. thermophila* D14 growth trials under different salinity, temperature, pH, and nitrogen source conditions, as well as from the biostimulant bioassay, was performed through one-way ANOVA with post hoc Tukey HSD, with Scheffé, Bonferroni, and Holm multiple comparison results was calculated for each condition on Astatsa.com (Vasavada 2016). A detailed presentation of the statistical results is provided in Supplementary Material B.

## Results

### Isolation and draft genome

Solar panel samples were collected during the summertime of 2013 and 2014 for screening cyanobacteria and microalgae. The first isolation was made in Castenholz-D medium (Baldanta et al. 2023) but microalgae and cyanobacteria were then maintained on BG11. At the beginning, a consortium among microorganisms, bacteria, and cyanobacteria/microalgae was evident on BG11 plates. After several streaks, different strains could be isolated (Baldanta et al. 2023). One of these strains, a unicellular green microalga, named D14, was initially identified by PCR 18S rRNA amplification, reaching a homology of 99% with other *Coelastrella* strains.

**Fig. 1:**
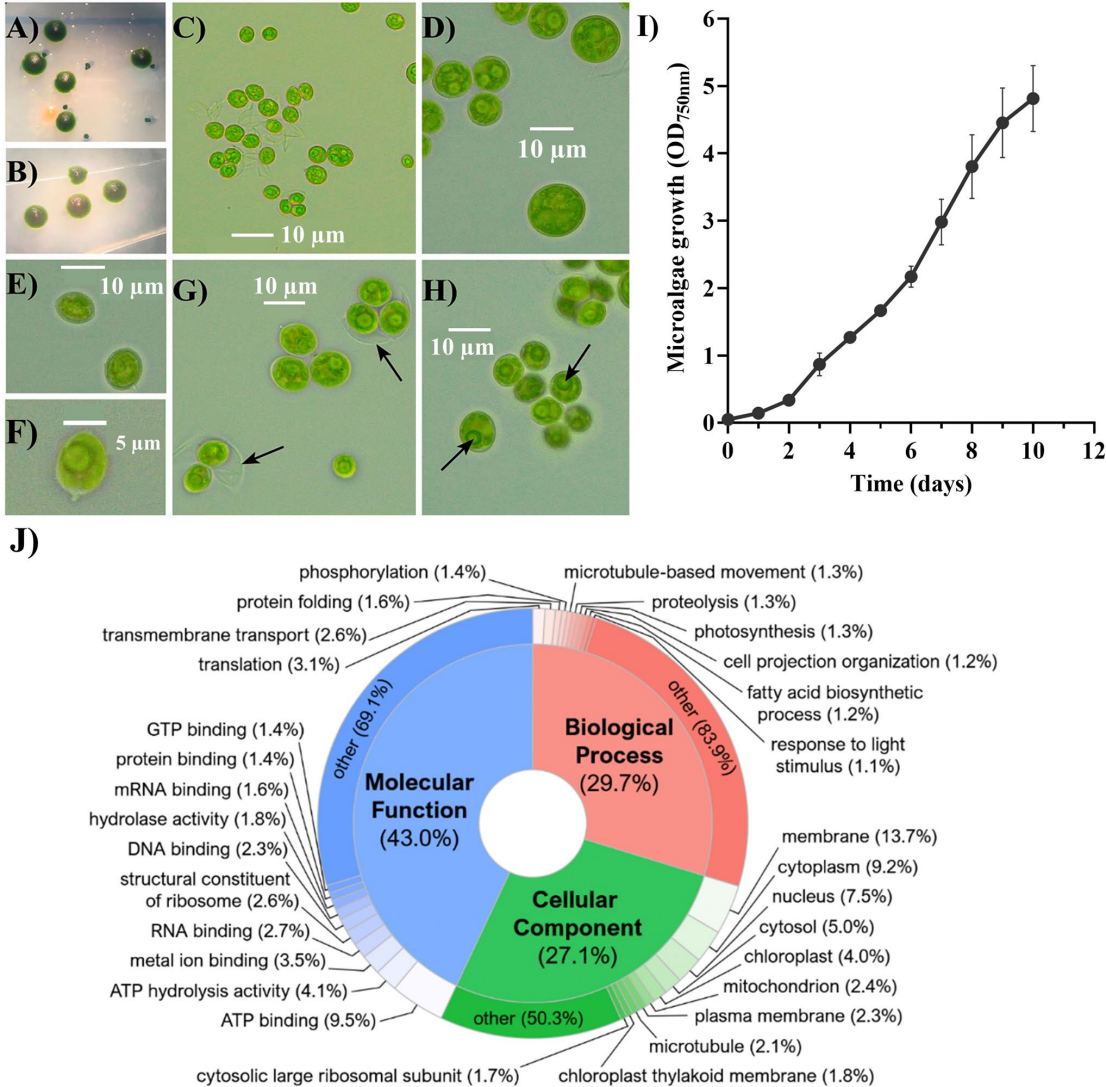
Isolation and characterization of *Coelastrella thermophila* D14. **A** Streak colonies from **A** the original consortium microalgae-bacteria and **B** the axenic microalgae. **C**–**H** Bright-field photomicrographs of *C. thermophila* D14 in BG11 on different days of culture: **C** young culture (3 days); **D** mature and round-shaped cells (10 days). **E**, **F** Lemon-shaped cells. In **F**, the arrow indicates a wart-like wall thickening. **G**, **H** Cells after division. In **G**, arrows indicate the cell wall of the mother cell and in **H**, the pyrenoids are pointed. Scale bar is shown in all the pictures. **I** Growth curve of *C. thermophila* D14 on BG11. Average OD_750nm_ ± standard deviation is depicted (*n* = *3*). **J** Description of the predicted proteins of *Coelastrella thermophila* D14 based on Gene Ontology terms. Each category (biological process, cellular component, and molecular function) shows its top ten most represented terms

Streaked microalgal colonies on agar plate, and microscopic observation is shown in Fig. 1A–H. Light microscopical observations showed that isolated *Coelastrella* D14 was a unicellular green coccoid microalga. The cells appeared as single oval cells, although a large degree of variation in cell size was observed ranging between 4.2 and 14.8 µm, with a mean diameter of 8.68 ± 1.96 μm. Single cells were smaller, had a lemon-shaped after division, a thin wall, and the pyrenoid was clearly noted. Also, in some of them, wart-like wall thickenings were observed (Fig. 1F). As the culture grew, the cells became round shaped and formed small groups of 2–6 cells. Figure 1G shows 2–3 daughter cells after cell division with the cell wall of the mother cell surrounding the new cells. In mature cells, the chloroplast is dissected into blades.

To investigate which species of *Coelastrella* was isolated (Baldanta et al. 2023), its nuclear genome has been preliminary sequenced. Approximately 1.3 million paired-end reads were retained after trimming and used for the assembly. The 60,874 initial contigs were assembled into 34,735 scaffolds (Table S1, Supplementary Material A). The final size of the draft genome was around 83 Mb, with an N50 value of 3568. Regarding the expected gene content of the genome, BUSCO results revealed 875 complete (57.6%), 311 fragmented (20.5%), 333 missing (21.9%), and 10 duplicated (0.7%) marker genes. The draft genome was deposited at https://zenodo.org/records/11631735 (10.5281/zenodo.11631734).

This preliminary genomic sequence yielded 3803 genes predicted (8545 exons and 6715 introns), of which 2032 were functionally annotated. Furthermore, the characteristics of the annotated proteins were summarized based on the three main Gene Ontology (GO) categories: biological process, cellular component, and molecular function. Figure 1J summarizes the ten most represented GO terms in each category out of the total 8550 different terms obtained in the analysis.

The pathways analysis revealed that the most represented ones in the functional annotation are related to metabolism, namely carbohydrate, energy and amino acid metabolism, and genetic information processing, namely transcription, translation and folding, sorting and degradation (Table 1).

**Table 1:** Most represented pathways in the *Coelastrella thermophila* D14 draft genome functional annotation

Pathway identifier	Pathway name	Number of genes
03010	Ribosome	81
03040	Spliceosome	27
00190	Oxidative phosphorylation	25
04141	Protein processing in endoplasmic reticulum	23
00195	Photosynthesis	21
00010	Glycolysis/gluconeogenesis	20
00620	Pyruvate metabolism	20
00270	Cysteine and methionine metabolism	19
00710	Carbon fixation in photosynthetic organisms	18
00970	Aminoacyl-tRNA biosynthesis	17

Thirty available sequences for the *Coelastrella* genus in the BOLD Systems database (Ratnasingham and Hebert 2007) were downloaded and Blastn was conducted against the assembled draft D14 genome. The only complete barcode sequence that produced a 100% match was that of *Coelastrella thermophila*, indicating, therefore, that the microalgae used in this study belongs to this species.

## Autotrophic growth

The growth evolution of *C. thermophila* D14 in BG11 medium (pH 7.5) for 10 days at 30 °C under 100 μE m^−2^ s^−1^ was studied (Fig. 1I, Table S2, Supplementary Material A). A mild lag phase of 2 days was observed and then, the microalga reached 2.60 ± 0.01 days of doubling time and an average productivity of 0.253 g L^−1^ day^−1^. The calculated correlation between the OD_750nm_ and cells per mL was as follows: Nº cells mL^−1^= 3.191 × 10^6^ OD_750_ (*R*^2^ = 0.99). In addition, the correlation of the biomass dry weight with OD_750nm_ was determined through gravimetry (Dry weight = 0.5314 × OD_750_−0.0127; *R*^2^ = 0.99).

**Fig. 2:**
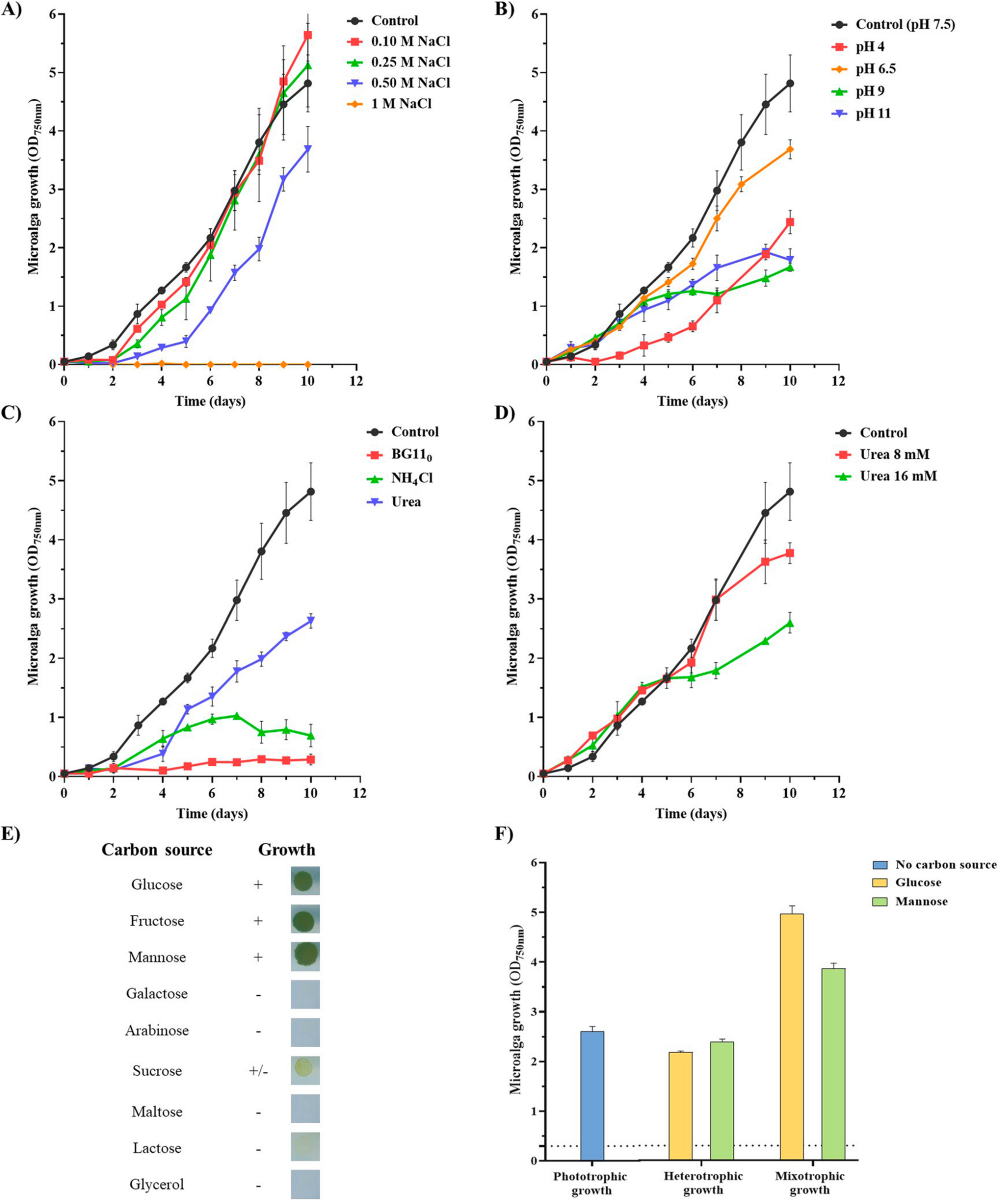
Growth characterization of *Coelastrella thermophila* D14 in different conditions. Autotrophic growth: **A** effect of NaCl concentration (0.1, 0.25, 0.5, and 1 M). **B** Effect of pH (4, 6.5, 9, and 11). **C** Effect of nitrogen source (urea and NH_4_Cl at 16 mM). In addition, BG11 with no nitrogen source was tested (BG11_0_). **D** *C. thermophila* D14 tolerance to urea (8 and 16 mM). For **A** to **D**, *C. thermophila* grown under routine conditions (BG11, 16 mM NaNO_3_ as nitrogen source, pH 7.5, 30 °C, 150 rpm, and continuous light 100 μE m^−2^ s^−1^) was used as control. Heterotrophic growth: **E** effect of different carbon sources (glucose, fructose, mannose, galactose, arabinose, sucrose, maltose, lactose, and glycerol). **F** Evaluation of phototrophic, heterotrophic, and mixotrophic growth in *C. thermophila* D14. OD_750nm_ was measured after 5 days of growth from an initial OD_750nm_ of 0.3 (dashed line). Phototrophic growth was performed in BG11, whether heterotrophic growth was tested by using the photosynthesis inhibitor DCMU (10 µM) and the selected carbohydrate. For mixotrophic growth, sugars were added but not DCMU. In all cases, the graphs show the average OD_750nm_ and error bars, the standard deviation (*n* = *3*). The conditions that shown a statistical difference compared to the control are depicted with *. One-way ANOVA with post hoc Tukey HSD, with Scheffé, Bonferroni, and Holm multiple comparison results was calculated for each condition. More information about the statistical analysis is summarized in Section B1 in Supplementary Material B

Regarding salt stress, *C. thermophila* D14 grew up to 0.5 M of NaCl (Fig. 2A, Table S2, Supplementary Material A). The growth was slightly improved at 0.1 M, 0.25 M, and 0.50 M of NaCl, according to the statistical analysis (Section B1, Supplementary Material B). However, at 0.5 M of NaCl, the lag phase was 3 days longer and the maximal OD was lower compared to control. No growth was observed at 1 M NaCl.

The best pH to grow *C. thermophila* D14 was 7.5 (control) as shown in Fig. 2B (Table S2, Supplementary Material A). Non-statistical differences were observed in the doubling time for pH 6.5 when compared to the control; however, a lower growth was reached after 10 days. Interestingly, despite having an extended lag phase, *C. thermophila* D14 grew at pH 4 with a doubling time, which was similar to that calculated for pH 7.5 (non-statistically different) after 5 days of growth. On the other hand, the microalga was able to tolerate pH 9 and 11, with a significantly higher doubling time and reaching a lower OD than the control (5.91 and 3.39 days, respectively vs. 2.57 days at pH 7.5) (Section B1, Supplementary Material B). The results demonstrate that *C. thermophila* D14 is broadly resistant to pH from 4 to 11. More specifically, in those cultures, the final pH was ~ 6.3 and ~ 9.5, respectively and the cultures remained green after the experiment (Fig. S3, Supplementary Material A).

Temperature greatly affected the growth of *C. thermophila* D14 (Fig. S3, Supplementary Material A). No growth was observed at 50 °C, and the cultures were bleached on the second day of the experiment. At 4 and 40 °C, we had a small increase in the microalga growth, and the cultures remained pale green, indicating they were highly stressed.

The effect of the absence or the use of different nitrogen sources at 16 mM (the same concentration as NaNO_3_ is used in BG11) for the cell growth was assessed in liquid cultures. Figure 2C shows the growth of the microalga over 10 days using alternative nitrogen sources, such as urea or ammonium. *C. thermophila* D14 could not grow without any nitrogen source, which may be because they lacked the ability to fix atmospheric nitrogen in the conditions tested. Growth in the presence of ammonium was also closer to the result observed in BG11, but only for the first 6 days. After that, the increase of OD stopped, giving a similar trend observed in BG11_0_. The results show that urea could also be used as a nitrogen source, with no statistical differences (Section B1, Supplementary Material B) and with a similar doubling time (2.52 days) compared to the control (with nitrate) but with a longer lag phase (4 days instead of 2 days) (Table S2, Supplementary Material A).

Considering that urea was the best alternative nitrogen source for growing the microalgae (Fig. 2C) and can be abundant in different types of wastewaters, various concentrations of urea were tested to grow *C. thermophila* D14 (Fig. 2D). The microalga was sensitive to 16 mM urea, while 8 mM allowed a better growth, although lower than the control at the end of the experiment, as the cells reduced the growth after 7 days of culture. These results show that even though urea cannot replace NaNO_3_ as a nitrogen source, some strains can tolerate it.

### Heterotrophic and mixotrophic growth

The growth of *C. thermophila* D14 was tested heterotrophically, as this strategy is widely used to increase biomass concentrations, especially to inoculate large-scale autotrophic cultures. First, growth was evaluated using glycerol and eight different sugars at 10 mM in BG11 in agar medium, complete darkness and with the photosynthesis inhibitor DCMU at 10 μM final concentration, for 30 days. The microalga was able to use glucose, fructose, and mannose as carbon source, but showed a weak growth using sucrose, and could not grow on maltose or lactose (Fig. 2E).

Erlenmeyer flask experiments were done to find accelerated mixotrophic biomass growth compared to an autotrophic condition. The carbon sources investigated were glucose and mannose as they contain the same amount of carbon (both are hexoses). Strictly heterotrophic growth was assured by adding DCMU to cultures with carbon source and light. Cultures without a carbon source represent phototrophic growth (control). As it is shown in Fig. 2F, the culture supplemented with glucose reached the highest OD_750nm_ compared to mannose (mixotrophic) and phototrophic growth. No differences were observed between the autotrophic and the heterotrophic growth for both carbon sources.

### Resistance to desiccation

**Fig. 3:**
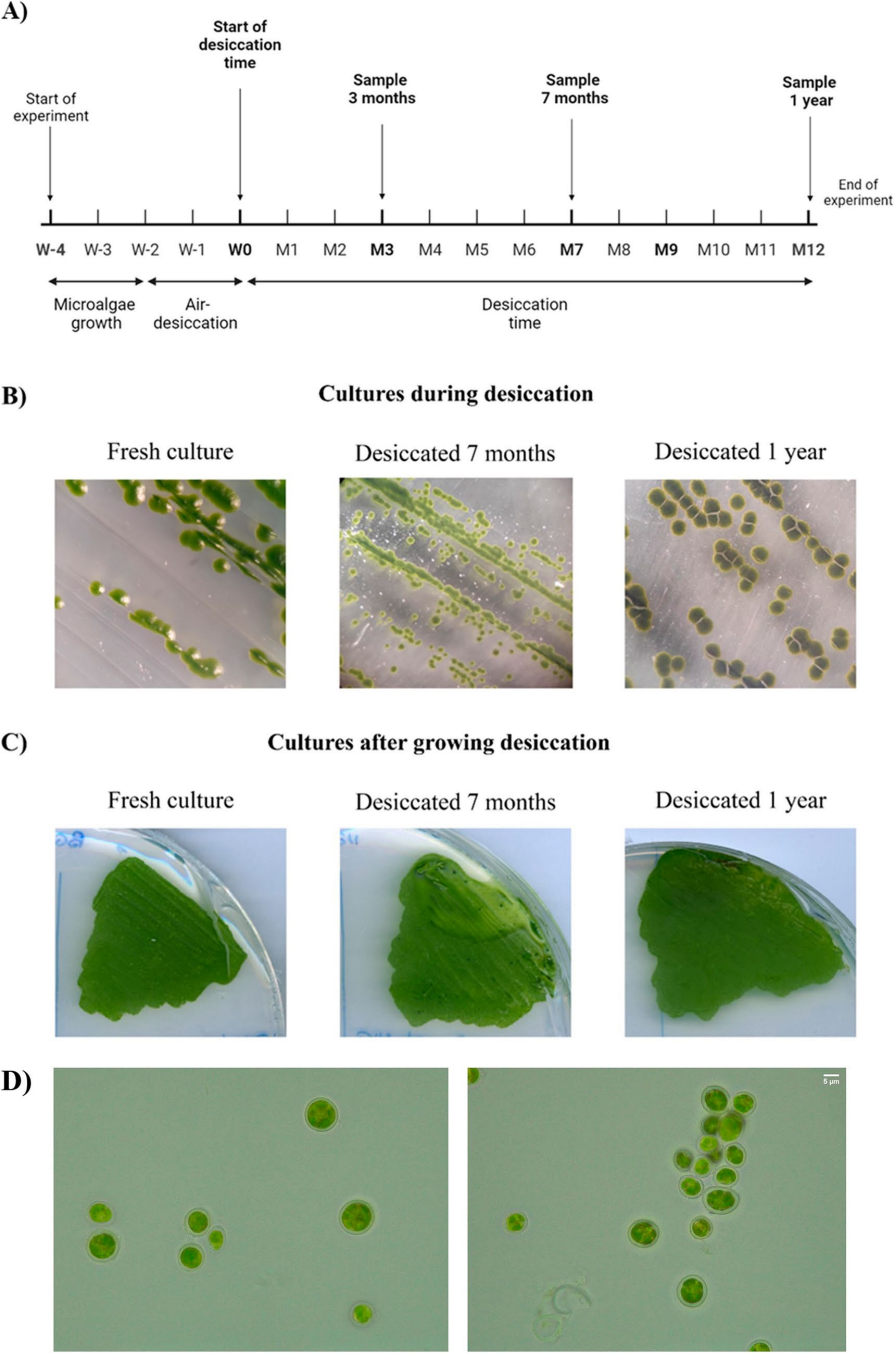
Long-term desiccation tolerance in *Coelastrella thermophila* D14. **A** Diagram of the desiccation process (M represents months). D14 cultures were grown for 2 weeks. Time 0 was considered to begin once the samples were dried, after 2 weeks. **B** Microphotographs of *C. thermophila* D14 during the experiment. **C** Growth observed after 2 weeks on BG11 post-rewetting for 7-month and 1-year desiccated culture. A freshly streaked strain was included as a control. **D** Light microscopical observation of *C. thermophila* D14 just after rewetting 7-month and 1-year desiccated samples

Considering that the microalga has been isolated from a solar panel, it was of interest to study its capacity to resist desiccation. Desiccation-tolerance tests were performed for 3 and 7 months, and 1 year (Fig. 3). As shown in Fig. 3A, in terms of ability to form colonies and grow, the rewetted samples differed little from the non-dried cultivated form, indicating that *C. thermophila* D14 was drought resistant. *Synechocystis* sp. PCC 6803, which was used as a non-resistant photosynthetic microorganism, was unable to grow after 3 months, the minimum time tested (data not shown). Just after the rewetting, all the *C. thermophila* cells exhibited the same morphology, with a thick sheath surrounding the cells (Fig. 3D).

## Microalgae growth in wastewater

The growth of *C. thermophila* D14 in wastewater was evaluated, more specifically in a piggery effluent, which composition was described previously.

**Fig. 4:**
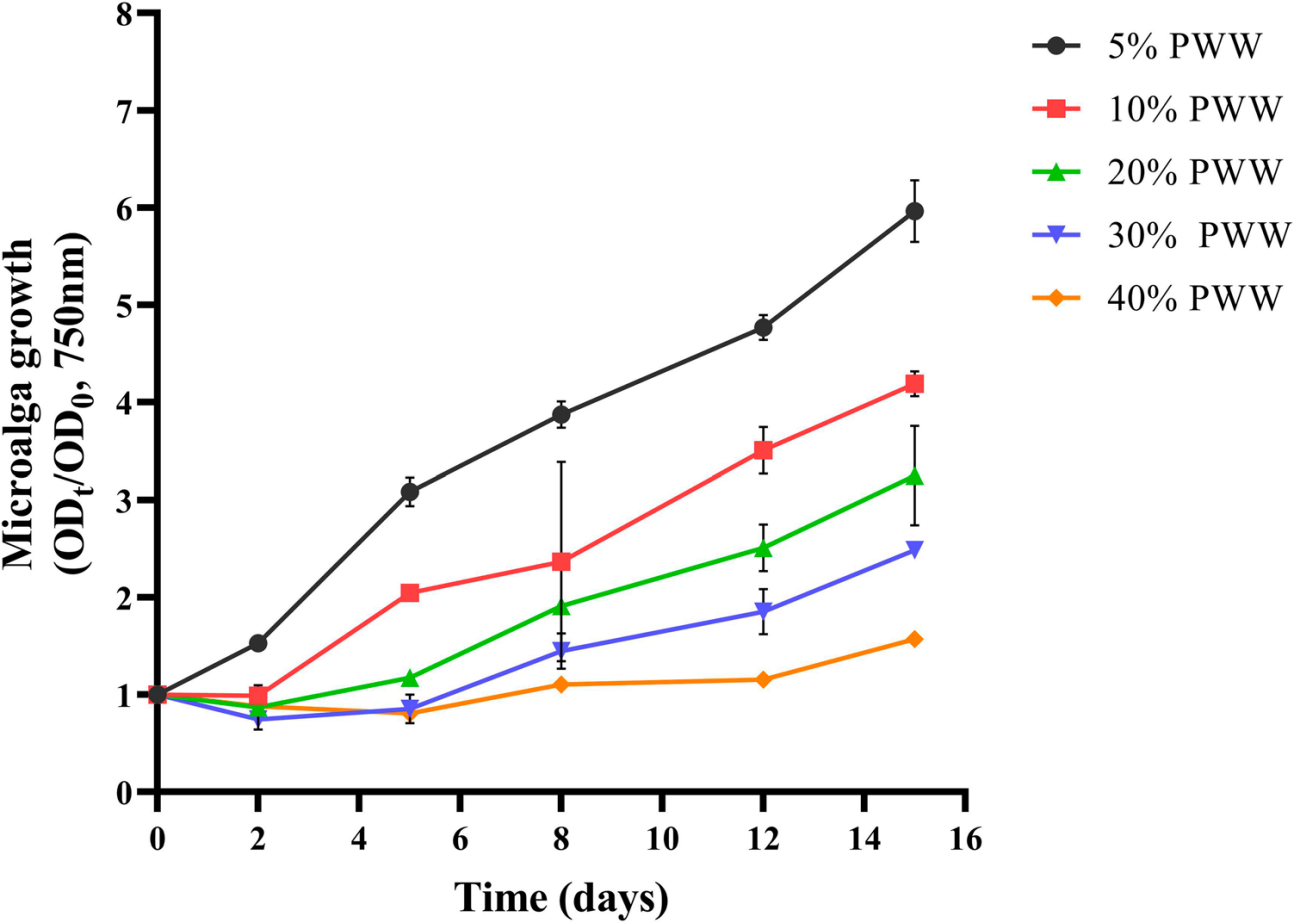
Growth of *Coelastrella thermophila* D14 at different concentrations of piggery wastewater (5, 10, 20, 30, and 40% v/v). In all cases, the graphs show the normalized value of the average OD_750nm_ together with the standard deviation (*n* = *2*)

*C. thermophila* D14 was submitted to an initial screening in different PWW concentrations (Fig. 4). Experiments demonstrated that D14 could grow in this effluent at concentrations up to 30% (v/v). However, the initial lag phase becomes longer with increasing PWW concentration. At 40% PWW, no visible growth was observed during the 15 days of the experiment. Native microorganisms could be found in the medium and co-cultivated with the inoculated D14. Nevertheless, in all experiments, the inoculated microalga rapidly outcompeted other organisms present in the wastewater. Thus, their impact on growth and biomass concentration was negligible.

### Biostimulant effect

**Fig. 5:**
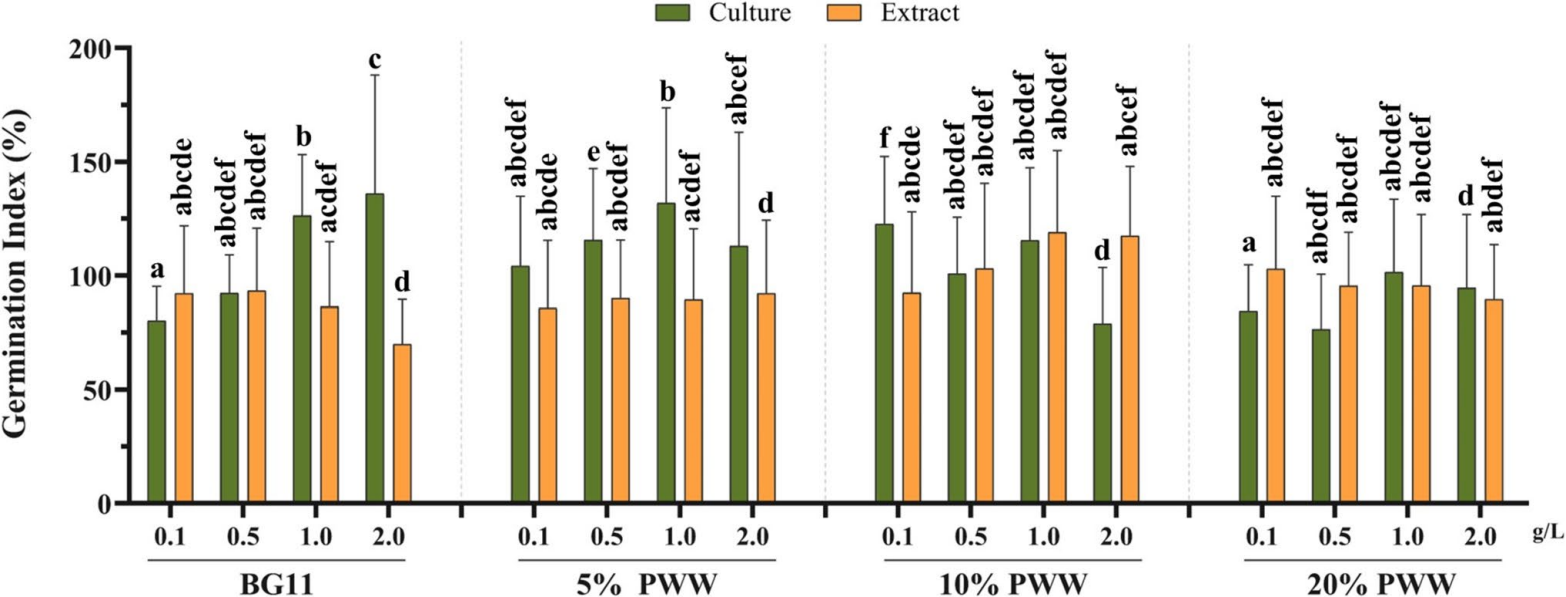
Germination index (%) of garden cress (*Lepidium sativum*) seeds treated with *Coelastrella thermophila* D14 biomass. The graph shows the mean values of germination index (GI), either using whole intact biomass or the broken biomass sample of D14 growth in four different media (BG11 or diluted (5, 10, and 20%) piggery wastewater, PWW). GI of 100% obtained when using distilled water as medium was considered as control. Error bars indicate standard deviation (*n* = *20*). Different letters indicate significant difference (*p* < 0.05) among treatments according to Tukey’s two-sided post hoc test. More information about the statistical analysis is summarized in Section B2 in Supplementary Material B

Plant growth is affected by phytohormones, amino acids, and polysaccharides, along with other nutrients, available from various sources, including microalgae. Here, the effect of *C. thermophila* D14 biomass was evaluated on germination of garden cress seeds (*Lepidium sativum*). In the biostimulant assay, a germination index (GI) of 100% was attributed to distilled water (control). Values higher than the control were considered to have a biostimulant activity (Fig. 5), and statistical analysis was performed (Section B2, Supplementary Material B). The highest GI values obtained were 132% at 1 g L^−1^culture on 5% PWW, followed by 128% corresponding to non-disrupted biomass trials at 1 or 2 g L^−1^ on BG11 medium. Cell disruption causes a statistically significant drop, for instance, at 2 g L^−1^ of D14 grown on BG11, yield dropped 45% of the GI value with respect to the whole biomass (Section B2, Supplementary Material B).

This tendency is not seen when it grew on 20% PWW. In this case, there are not so many differences between both cultures and broken-cells GI values, not reaching in any case the GI 100% value. In general, values under 100% may suggest that the microalga concentration or their biochemical composition might be excessive or toxic for *L. sativum*, negatively affecting their growth (Navarro-López et al. 2020).

Therefore, these results highlighted that 1 g/L of whole algal suspension grown at 5% PWW may be the best treatment for germination. However, the whole D14 biomass at 0.1 g/L grown in 10% PWW also yielded a statistically significant GI value compared to the control, specifically ∼23% higher than BG11. The latter is more interesting since it uses a higher percentage of PWW to grow, lower amount of non-processed biomass, decreasing downstream costs.

## Genetic transformation

The sensitivity to the antibiotics required for transformation was first determined (Table S3, Supplementary Material A). *Agrobacterium* was sensitive to cefotaxime starting from a concentration of 50 mg L^−1^, as previously reported (Cha et al. 2012), while *C. thermophila* D14 was resistant to cefotaxime 500 mg L^−1^. Therefore, to ensure complete elimination of *Agrobacterium* after co-cultivation, 500 mg L^−1^ was the concentration chosen for the transformation assays. On the other hand, *C. thermophila* D14 did not grow at any of the tested concentrations of hygromycin B (10, 20, 50 mg L^−1^); hence, 20 mg L^−1^ was used for the transformation assays.

**Fig. 6:**
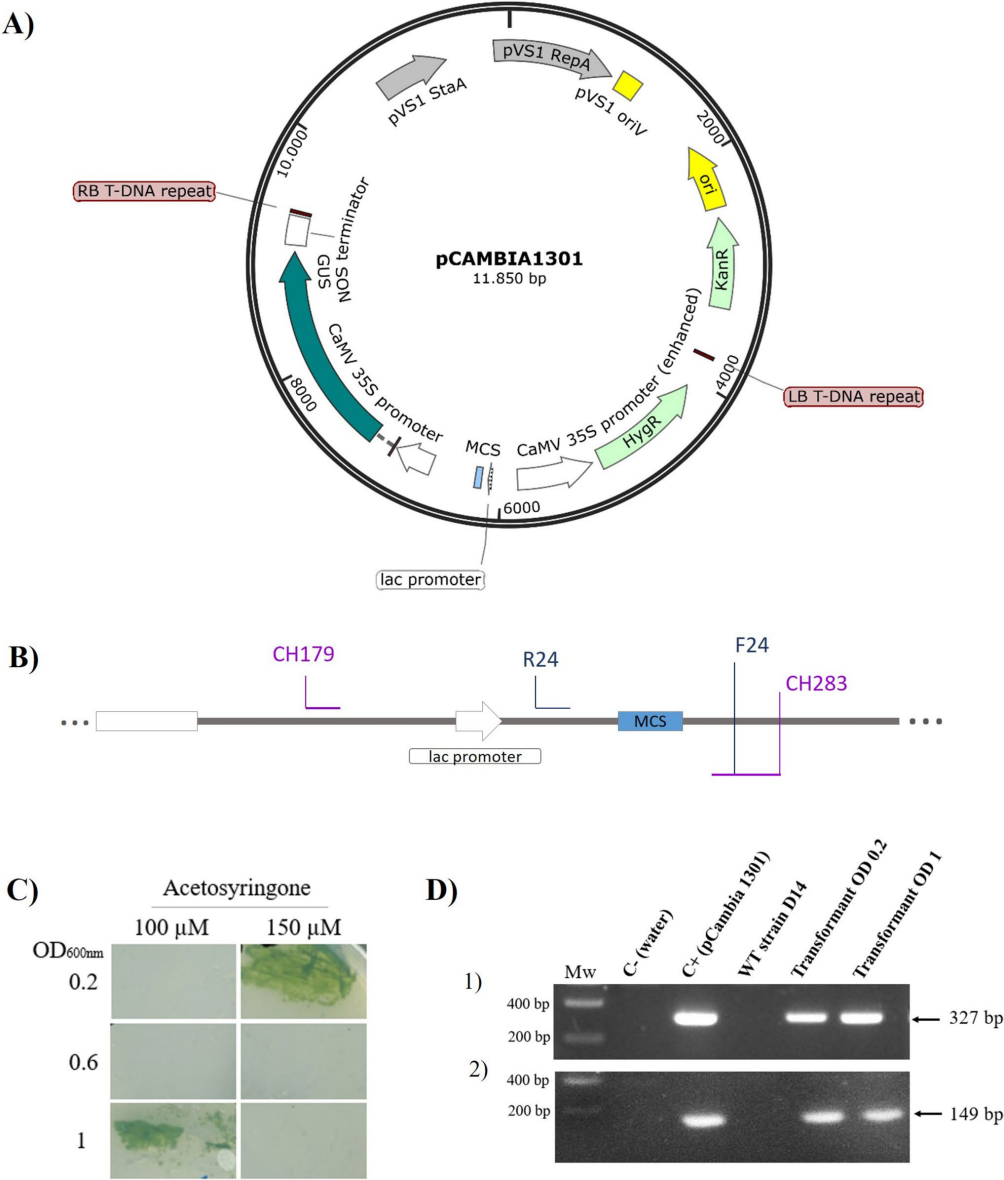
*Agrobacterium* mediated-genetic transformation of *Coelastrella thermophila* D14. **A** T-DNA from pCAMBIA 1301. **B** *C. thermophila* D14 transformed with pCAMBIA 1301 colonies growing in plates containing 20 mg L^−1^ hygromycin B; **C** PCR analysis of *C. thermophila* D14 transformants with T-DNA integrated into the genome. (1) Upper gel: amplification of the 327-bp fragment of the plasmid (CH283 and CH179 primers). (2) Bottom gel: amplification of the 149 bp of a second fragment with F24/R24 primers. Mw: 10-kb molecular weight marker

Using the pCAMBIA 1301 plasmid for all the transformation experiments (Fig. 6A, Fig. S2, Supplementary Material A), the influence of various factors known to affect *Agrobacterium*-mediated transformation efficiency was examined based on (Cha et al. 2012): bacterial density (OD_600nm_: 0.2, 0.6, or 1.0); duration of pre-culture (0 or 5 days); co-cultivation period (3 or 5 days); pH of the co-cultivation medium (5.0, 5.5, or 6.0); and acetosyringone concentration (100, 150, or 200 µM). To investigate the impact of each factor on transformation frequency, one factor was altered while keeping other factors constant (Fig. S2, Supplementary Material A).

Transformed colonies grew 15 days after co-cultivation (Fig. 6B). Only cells transformed directly from liquid media which were co-cultivated for 5 days at pH = 5.5 were successfully transformed. From all the trials, the final combinations that were successful getting recombinants able to grow in the selective media were as follows: (i) acetosyringone 150 µM, using an *Agrobacterium* of OD_600nm_ 0.2, and (ii) acetosyringone 100 µM with an *Agrobacterium* of OD_600nm_ 1.0 and co-cultivating strains for 5 days in both cases (Fig. 6B).

## Discussion

The isolate obtained from the solar panel was identified as a *Coelastrella thermophila* strain based on morphological characteristics and genomic analysis and subsequently renamed *C. thermophila* D14. The morphological traits of this strain (single oval cells with a mean diameter of 8.68 ± 1.96 μm) align with those typical of the genus *Coelastrella*, which is characterized by unicellular organisms that occasionally form aggregates, with vegetative cells ranging from spherical to subspherical and measuring 4.2–14.8 µm in diameter (Doppler et al. 2022; Shetty et al. 2021).

There are up to date three *Coelastrella* genomes sequenced in the NCIB database (GCA_0180290735.1: *Coelastrella* sp. MACC-549 75.79 Mb with 51.5% GC; GCA_001630525.1: *Coelastrella* sp. M60 80.2 Mb with 52% GC; and GCA_002588565.1: *Coelastrella* sp. UTEX B 3026 151.3 Mb with 52% GC content). The D14 draft genome displayed similar parameters, with a size of 83 Mb and 51.8% GC content. The D14 preliminary draft genome was used for species identification. A blastn with the whole draft genome using the BoldSystems platform against all the *Coelastrella* sequences was done as it has been reported that 18S rDNA was too much conserved to be used a species-specific or even a genus-specific marker in this clade (Wang et al. 2019b). Attending to its draft genome, this novel strain belongs to the Chlorophyceae class, Sphaeropleales order, Scenedesmaceae family, *Coelastrella* genus, and *Coelastrella thermophila*. Many species of the *Coelastrella* genus are isolated from harsh environments, for instance, Ki4 from a Japanese asphalt surface in midsummer (Kawasaki et al. 2020) or *Coelastrella* sp. isolate 3 A isolated from an Algerian hot spring (Boutarfa et al. 2022). Some of them are reported to display remarkable properties such as for producing n-6 and n-3 PUFA fatty acids (Boutarfa et al. 2022), for producing astaxanthin (Kawasaki et al. 2020) or for Bisphenol A degradation (Ajithkumar et al. 2024).

In the conditions tested, D14 displayed a doubling time of 2.6 days (Table S2, Supplementary Material A). A novel freshwater *Coelastrella* strain isolated in Belgium, presented a rapid phototrophic growth, with a doubling-time of 6.8 ± 0.30 h at a light intensity of 400 µE m^−2^ s^−1^ and 5% CO_2_ (Corato et al. 2022). The specific growth rate seems to depend, among other factors, on dosing times of the carbonic solution added to the culture. This causes an increase on the lipids and proteins content with major carbon source dosing times, while the carbohydrate content decreases, suggesting that the carbon source is a critical parameter for algal growth (Razooki et al. 2019). The doubling time of D14 is given without any extra C source, which could explain the low value obtained and leaves room for improvement by increasing the percentage of CO_2_ or adjusting the amount of light during growth.

D14 growth was assayed under several conditions as microalgal growth and biomass production depends on nutrient availability, light intensities and regime, pH or temperature, among others. Most microalgae can grow over the range of pH values from 6.8 to 8.0, the suitable pH value depending on the microalgal species (Daneshvar et al. 2021). Concretely, a range from 5.0 to 9.0 is reported for *Coelastrella* KKU-P1 strain (Thepsuthammarat et al. 2023). D14 grown in flasks is viable from a wide range of pH, from 4.0 to 11.0, although the highest production was obtained under a pH of 7.5 (Fig. 2). *Coelastrella* sp. strain D3–1 has been reported to resist from pH 2 to pH 11, but the experiments were performed in different conditions to this work. The cells were grown on BG11 plates for 7 days after the stress treatment on a diluted medium 0.2XBG11 (Saito et al. 2023), while in this work, data was taken in liquid medium with the adjusted pH. Regarding the temperature, it greatly affected the growth of *C. thermophila* D14 (Fig. S3, Supplementary Material A) as no growth was observed over 50 °C. However, several *Coelastrella* have been reported to be able to grow up to 50 °C (Saito et al. 2023) or even over (Nayana et al. 2022).

D14 was able to resist desiccation for up to 1 year (the maximum time tried). There are no similar experiments done with other *Coelastrella*. Still, a heat-dry stress done in *Coelastrella* sp. D3–1, in which cell pellet was exposed to 42 °C for 3 h in a dryer, showed that the microalga was able to grow when later was spotted on BG11 plates (Saito et al. 2023). The xerotolerance that D14 shown is consistent with its original habitat, a solar panel, in which the water activity is logically low. It is quite possible that other *Coelastrella* isolated from solid environments such that characterized from an asphalt surface (Kawasaki et al. 2020) could display a similar behavior. An ancestral origin of desiccation tolerance has been suggested due to the similar physiological response and the finding of shared molecular mechanisms to this process among photosynthetic organisms (Peredo and Cardon 2020). A change in expression of several genes has been reported to be involved in the stress tolerance of algae that also depends on the strain and kind of stress (Calhoun et al. 2021; Xing et al. 2022; Zhou et al. 2023). Some of the upregulated genes are the late embryogenesis abundant (LEA) proteins, components of biosynthetic pathways for oligosaccharide osmolytes, and scavengers for reactive oxygen species (ROS) (Holzinger et al. 2014; Kijak and Ratajczak 2020; Rippin et al. 2017). However, there is limited information available on drought resistance genes in microalgae. On the other hand, it has been reported a transcriptomic comparison among desiccation-tolerant (desert-derived) versus intolerant (aquatic) microalgae (Peredo and Cardon 2020), showing that during the desiccation process, there is both an upregulation of genes coding for protective proteins that it is also shared with the desiccation-intolerant strains, and an extensive downregulation of diverse metabolic genes (Peredo and Cardon 2020).

Several of the genes upregulated or downregulated, found in the transcriptomic study done by Peredo and Cardon (2020), can be found in the D14 draft genome (Supplementary Material C). For instance, there are up to 20 heat shock proteins in the D14 genome. These genes were specifically upregulated during the drought process of the desiccation-tolerant strains (Peredo and Cardon 2020). These proteins protect cells from adverse effects of thermal stress through chaperoning and refolding of polypeptides and by stabilizing protein complexes, cellular membranes, and key cellular processes. On the other hand, the D14 genome contains 22 chlorophyll a/b binding proteins-coding genes and 33 photosystem I subunits genes. Both kinds of genes were reported to specifically be downregulated during the dessication process (Peredo and Cardon 2020). However, as several studies have pointed out, it is necessary to use differential expression assays, such as transcriptomics, to determine the mechanisms of desiccation resistance.

To enhance biomass production, the growth of *C. thermophila* D14 was investigated under both heterotrophic and mixotrophic conditions. The microalga was able to grow well heterotrophically using glucose, fructose, and mannose as carbon sources, hardly with sucrose and could not grow on maltose or lactose (Fig. 2E). Some of the metabolic genes that could be found in the D14 draft genome related to metabolism can be found at Supplementary Material D. In mixotrophic conditions, the growth of D14 reached the highest OD_750nm_ when the medium was supplemented with glucose, in comparison to the use of mannose or phototrophic growth. In general, green microalgae can efficiently use glucose and fructose for growing but they usually lack sucrose transporter systems (Pang et al. 2019). However, there are some reports in which *Coelastrella* sp. KKU-P1 is capable of sucrose consumption and could be used for growing on unhydrolyzed molasses as a low-cost carbon source that is rich in sugars, mainly sucrose, glucose, and fructose (Thepsuthammarat et al. 2023). D14 could be subjected to adaptive laboratory evolution experiments to improve its mild growth on sucrose and in this way, being able to grow on more low-cost substrates.

In autotrophic conditions, D14 was not able to survive int the absence of nitrogen in the medium (Fig. 2C). This is expected as nitrogen is an essential component for microalgae growth, needed for macromolecules synthesis, among other things, and *Coelastrella* was not able to fix N_2_, at least in the conditions tested. The microalga was able to grow in urea or NH_4_Cl (16 mM) as the nitrogen source, but not as well as the original nitrate source. Ammonium is the usual preferred nitrogen source for algae since it consumes less energy, as it does not require a redox reaction (Nayana et al. 2022). Some of the metabolic genes related to nitrogen metabolism could be found in the D14 draft genome (Supplementary Material D).

The use of low-cost sources of nitrogen, such as urea, is convenient for reducing economical costs of microalgae growth. Due to the ability of microalgae to grow in very diverse environments, and considering the idea of circular economy, wastewaters that are rich in pollutants that can be used as a culture medium. In fact, growing microalgae in wastewater is a suitable alternative to reduce freshwater expenses and valorize residual nutrients (Ahmed et al. 2022; Sánchez-Quintero et al. 2023). Sharma et al. (2022) enumerate several economic and growth considerations when cultivating microalgae in wastewater pointing to that appropriate strains’ selection is crucial for the whole process. Therefore, it is important to count on different strains or consortiums able to bioremediate the effluents and being useful for interesting applications.

One type of wastewater that raises significant concerns is that generated by the piggery industry, characterized by its complexity and high nutrient content, including ammonia and organic matter and its deep dark color impedes light penetration, affecting photosynthesis (Ferreira et al. 2021). This effluent contributes to eutrophication and freshwater ecosystem toxicity when released untreated into rivers (Ferreira et al. 2021; Lee et al. 2021; Li et al. 2019). In addition, direct utilization of piggery effluents in agricultural composting leads to the generation of greenhouse gas emissions, such as CO_2_ and N_2_O (Hu et al. 2020; Mohedano et al. 2019), soil, and deep-water pollutions. Consequently, numerous efforts have been made to explore biological treatment options for raw PWW using microalgae, employing various strategies.

Pilot-scale studies treating undiluted raw PWW with microalgae have demonstrated efficient nutrient removal and improved wastewater clarity (Lee et al. 2022). In another approach, *Coelastrella* sp. isolated from an ammonia-rich environment was used for PWW treatment in a 4-day two-step process: heterotrophic plus mixotrophic steps in a narrow transparent photobioreactor (Lee et al. 2021). Under these conditions, *Coelastrella* sp. achieved remarkable removal efficiencies, removing 99% of ammonia, 92% of COD, and 100% of phosphorus. Notably, in this instance, the microalgal biomass was directed towards high-quality biodiesel production (Su et al. 2023). The present study highlights that strain D14 can thrive in piggery wastewater concentrations up to 20%. However, further investigations are warranted to assess potential alterations in the biochemical composition of the microalga under such conditions.

In this work, besides assessing the potential of *C. thermophila* D14 to grow in piggery wastewater, the use of the resulting biomass was explored to stimulate plant growth. A bioassay was performed based on the germination index of *Lepidium sativum* seeds. The results have shown that D14 has potential as a biostimulant product, showing a gibberellin-like effect when grown on BG11, applied at 1–2 g L^−1^ or 5% PWW, applied at 1 g L^−1^, yielding GI values up to 132%. Other bioassays in agricultural models, such as examining root formation in mung beans and cucumbers, will be necessary to further demonstrate the auxin and cytokine-like effects. On the other hand, data obtained with the whole biomass yielded higher GI values with respect to the broken cells. For other strains such as the microalga *Scenedesmus obliquus*, a similar behavior has been reported. For this microalga, grown on brewery effluents, a GI of 139% was obtained (Navarro-López et al. 2020).

There are recent strategies of culturing microalgae using wastewater and CO_2_ to produce large quantities of biomass at moderate costs while integrating local and circular economy approaches (Sánchez-Quintero et al. 2023). The fact that the microalgal D14 biomass could be used directly as a biostimulant implies a reduction in economic costs and a sustainable application, avoiding synthetic ones. Regarding the use of raw piggery wastewater, despite that microalgal treatment reduces the risk of pathogens by 63% (Lee et al. 2022), recent reports highlight their persistence. To align with EU Regulation 2019/1009 on plant biostimulants, it is recommended to use broken-cells to mitigate potential pathogen presence (Lee et al. 2022; Sánchez-Quintero et al. 2023). Further optimization studies are needed on the preparation of alga extracts, as stated procedures may impact the final bioavailability of microalga compounds. These factors are influenced by the microalga species, cultivation medium, and culture state (Ferreira et al. 2018, 2019). More specifically, bioactive compounds reported as biostimulants are phytohormones, heteropolysaccharides, amino acids, or vitamins (as reviewed by Sánchez-Quintero et al. (2023)), which are produced in various phases of growth (Tan et al. 2021). This variability could explain why the broken-cell samples with the same concentration but grown in different media (resulting in distinct growth curves) do not exhibit the same biostimulant behavior.

Due to diversity of microalgae, it is necessary to count on customized molecular tools for each specific host as being able to be transformed is an essential prerequisite to biotechnological applications (Gutiérrez and Lauersen 2021). Since the first microalgae gene transformation in *Chlamydomonas reinhardtii* in 1982 (Rochaix and van Dillewijn 1982), there is still a lack of universally applicable transformation methods in most microalgal strains (Kuo et al. 2022). The gene transformation method mediated by *Agrobacterium* harboring engineered tumor-inducing (Ti) plasmid has been applied for efficient gene transfer in several microalgae such *Chlorella*, *Dunaliella*, *Chlamydomonas*, or *Nannochloropsis* sp. (Kuo et al. 2022; Sanitha et al. 2014). Therefore, this method was tested as an alternative method for D14, as previous attempts with electroporation failed (data not shown). The efficiency of *Agrobacterium*-mediated gene transformation in microalgae is reported to be affected by several factors such as the bacterium density, temperature, pH, and time of co-cultivation, as well as the supplement of an infection inducer, acetosyringone (Cha et al. 2012; Kuo et al. 2022). Therefore, several transformation conditions were probed to find the best combination. For the transformation experiments, pCAMBIA 1301 vector, an *Agrobacterium* binary vector for plant transformation, with hygromycin and GUS genes, was chosen, as it has been used for *Chlorella*, *Scenedesmus bajacalifornicus*, and *Ankistrodesmus* sp. (Sanitha et al. 2014). D14 transformation was successful, and gene integration was confirmed by PCR amplification of the *lacZ* gene. Unlike the microalga *C. vulgaris* (Cha et al. 2012), *C. thermophila* D14 did not need to be cultured on solid media to achieve T-DNA transfer. In fact, plate culture may not yield positive results for this microalga. Another important factor is the co-cultivation time, as this microalga takes longer to grow and therefore requires more days of co-cultivation with *Agrobacterium*. This is the first report of a *Coelastrella* strain to be transformed.

This work opens avenues for future studies, including the optimization of the *Agrobacterium*-mediated transformation protocol for *C. thermophila* D14. Another important step involves developing a high-quality reference genome sequence of *Coelastrella* coupled with transcriptomic studies, such as RNA-seq, that could provide valuable insights into the genetic regulation of adaptive traits, facilitating the understanding and manipulation of metabolic pathways to produce high-value compounds ranging from biofuels to industrially relevant substances. Moreover, future research could explore the potential of D14 as a renewable source of food and animal feed, as well as its capacity for energy production through bioconversion processes. Additionally, investigating its role in CO2 capture and conversion could further enhance its application in sustainable practices. The demonstrated biostimulant properties of *C. thermophila* D14 warrant further exploration through large-scale field trials in diverse agricultural contexts. Finally, assessing the scalability and economic viability of utilizing this strain in industrial applications, particularly in sustainable agriculture and wastewater management, will be essential for realizing its full biotechnological potential.

## Conclusions

Microalgae hold immense promise as a feedstock for producing high-value products. Yet, the key to unlocking their full potential lies in isolating strains that thrive under stressful conditions. This approach not only minimizes contamination but also ensures a unique biochemical profile, broadening their biotechnological applications. Our research highlights *Coelastrella thermophila* D14, a strain capable of flourishing on cost-effective substrates like piggery wastewater (PWW) and exhibiting resilience to prolonged desiccation periods. Notably, when grown at 5% PWW, the biomass of D14 can serve as a biostimulant, paving the way for more sustainable practices. Furthermore, the genetic transformability of *C. thermophila* D14 opens new opportunities for genetic engineering, enhancing its utility in various biotechnological innovations.

## Supplementary information

Below is the link to the electronic supplementary material.

ESM1 (DOCX 833 KB)

ESM2 (DOCX 40.1 KB)

ESM3 (XLSX 21.0 KB)

ESM4 (XLSX 21.0 KB)

## Data Availability

The draft genome was deposited at https://zenodo.org/records/11631735 (DOI: 10.5281/zenodo.11631734). Other data will be made available on supplementary material.
