# Supplementary material for: A solar panel-origin microalga, Coelastrella thermophila D14, with high potential for wastewater biotechnology
Source: Appl Microbiol Biotechnol. 2025 Nov 24;109:246. doi: 10.1007/s00253-025-13618-8 (PMC12647267; doi:10.1007/s00253-025-13618-8)
Supplement: Supplementary file 2 [file 253_2025_13618_MOESM2_ESM.docx]

**Supplementary Material B. Statistical Analysis**

**B1.** One-way ANOVA with post-hoc analysis of doubling time calculations for *Coelastrella thermophila* D14 in several growth conditions

**ANOVA: SINGLE FACTOR for NaCl**

**Summary**

| **Groups** | **Count** | **Sum** | **Average** | **Variance** |
| --- | --- | --- | --- | --- |
| Normal | 4 | 10.26420338 | 2.566050845 | 0.01574219 |
| 0.1 M | 3 | 5.970823556 | 1.990274519 | 0.01185347 |
| 0.25 M | 3 | 4.617741296 | 1.539247099 | 0.008428947 |
| 0.50 M | 3 | 4.812854545 | 1.604284848 | 0.009295668 |

**ANOVA**

| **Source of Variation** | **SS** | **df** | **MS** | **F** | **P-value** |
| --- | --- | --- | --- | --- | --- |
| Between Groups | 2.38 | 3 | 0.7933 | 67.12 | 1.76E-02 |
| Within Groups | 0.11 | 9 | 0.0118 |  |  |
| Total | 2.49 | 12 |  |  |  |

H0:	Normal=0.1M=0.25M=0.5M (NaCl)

If p<0.05, we reject the null hypothesis, meaning at least one condition is different.

The p-value corresponding to the F-statistic of one-way ANOVA is lower than 0.05, **suggesting that one or more treatments are significantly different**. The Tukey HSD test, Scheffé, Bonferroni and Holm multiple comparison tests follow. These post-hoc tests would likely identify which of the pairs of treatments are significantly different from each other.

**Tukey HSD Test**

| **Treatments pair** | **Tukey HSD Q statistic** | **Tukey HSD p-value** | **Tukey HSD inference** |
| --- | --- | --- | --- |
| Normal vs 0.1M | 9.8061 | 0.0010053 | ** p<0.01 |
| Normal vs 0.25M | 17.4876 | 0.0010053 | ** p<0.01 |
| Normal vs 0.5M | 16.3799 | 0.0010053 | ** p<0.01 |
| 0.1M vs 0.25M | 7.1854 | 0.0030256 | ** p<0.01 |
| 0.1M vs 0.5M | 6.1492 | 0.0082448 | ** p<0.01 |
| 0.25M vs 0.5M | 1.0361 | 0.8754074 | ns |

**Scheffé multiple comparison**

| **Treatments pair** | **Scheffé T-statistic** | **Scheffé p-value** | **Scheffé inference** |
| --- | --- | --- | --- |
| Normal vs 0.1M | 6.934 | 0.0005925 | ** p<0.01 |
| Normal vs 0.25M | 12.366 | 5.69E-02 | ** p<0.01 |
| Normal vs 0.5M | 11.582 | 9.87E-02 | ** p<0.01 |
| 0.1M vs 0.25M | 5.081 | 0.0052144 | ** p<0.01 |
| 0.1M vs 0.5M | 4.348 | 0.0136281 | * p<0.05 |
| 0.25M vs 0.5M | 0.7327 | 0.9080160 | ns |

**Bonferroni and Holm multiple comparison**

| **Treatments pair** | **Bonferroni and Holm**  **T-statistic** | **Bonferroni**  **p-value** | **Bonferroni inference** | **Holm p-value** | **Holm inference** |
| --- | --- | --- | --- | --- | --- |
| Normal vs 0.1M | 6.934 | 0.0004083 | ** p<0.01 | 0.0002722 | ** p<0.01 |
| Normal vs 0.25M | 12.366 | 3.5763E-06 | ** p<0.01 | 0.035763 | ** p<0.01 |
| Normal vs 0.5M | 11.582 | 6.2436E-06 | ** p<0.01 | 0.05203 | ** p<0.01 |
| 0.1M vs 0.25M | 5.081 | 0.0039726 | ** p<0.01 | 0.0019863 | ** p<0.01 |
| 0.1M vs 0.5M | 4.348 | 0.0111311 | * p<0.05 | 0.0037104 | ** p<0.01 |
| 0.25M vs 0.5M | 0.7327 | 2.8944934 | ns | 0.4824156 | ns |

ns: not significant

**ANOVA: SINGLE FACTOR for pH**

**Summary**

| **Groups** | **Count** | **Sum** | **Average** | **Variance** |
| --- | --- | --- | --- | --- |
| Normal | 4 | 10.26 | 2.57 | 0.02 |
| pH 4 | 2 | 4.18 | 2.09 | 0.03 |
| pH 6.5 | 3 | 7.018 | 2.34 | 0.01 |
| pH 9 | 3 | 17.73 | 5.91 | 0.10 |
| pH 11 | 3 | 10.17 | 3.39 | 0.14 |

**ANOVA**

| **Source of Variation** | **SS** | **df** | **MS** | **F** | **P-value** |
| --- | --- | --- | --- | --- | --- |
| Between Groups | 28.31 | 4 | 7.08 | 124.54 | 1.74E-08 |
| Within Groups | 0.57 | 10 | 0.068 |  |  |
| Total | 28.88 | 14 |  |  |  |

H0:	Control=pH6.5=pH9=pH4=pH11

If p<0.05, we reject the null hypothesis, meaning at least one condition is different.

The p-value corresponding to the F-statistic of one-way ANOVA is lower than 0.05, **suggesting that one or more treatments are significantly different**. The Tukey HSD test, Scheffé, Bonferroni and Holm multiple comparison tests follow. These post-hoc tests would likely identify which of the pairs of treatments are significantly different from each other.

**Tukey HSD results**

| **Treatments pair** | **Tukey HSD Q statistic** | **Tukey HSD p-value** | **Tukey HSD inference** |
| --- | --- | --- | --- |
| Normal vs pH 4 | 3.2544 | 0.2213706 | ns |
| Normal vs pH 6.5 | 1.778 | 0.7031955 | ns |
| Normal vs pH 9 | 25.9698 | 0.0010053 | ** p<0.01 |
| Normal vs pH 11 | 6.3943 | 0.0076264 | ** p<0.01 |
| pH 4 vs pH 6.5 | 1.5998 | 0.7679478 | ns |
| pH 4 vs pH 9 | 24.8153 | 0.0010053 | ** p<0.01 |
| pH 4 vs pH 11 | 0.84372 | 0.0010140 | ** p<0.01 |
| pH 6.5 vs pH 9 | 25.9557 | 0.0010053 | ** p<0.01 |
| pH 6.5 vs pH 11 | 7.6445 | 0.0021516 | ** p<0.01 |
| pH 9 vs pH 11 | 18.3112 | 0.0010053 | ** p<0.01 |

**Scheffé results**

| **Treatments pair** | **Scheffé T-statistic** | **Scheffé p-value** | **Scheffé inference** |
| --- | --- | --- | --- |
| Normal vs pH 4 | 2.3012 | 0.3262211 | ns |
| Normal vs pH 6.5 | 1.2572 | 0.8077480 | ns |
| Normal vs pH 9 | 18.3634 | 0.0011604 | ** p<0.01 |
| Normal vs pH 11 | 4.5214 | 0.0166641 | * p<0.05 |
| pH 4 vs pH 6.5 | 1.1312 | 0.8583344 | ns |
| pH 4 vs pH 9 | 17.547 | 0.0017996 | ** p<0.01 |
| pH 4 vs pH 11 | 5.966 | 0.0024888 | ** p<0.01 |
| pH 6.5 vs pH 9 | 18.3534 | 0.0011665 | ** p<0.01 |
| pH 6.5 vs pH 11 | 5.4055 | 0.0050924 | ** p<0.01 |
| pH 9 vs pH 11 | 12.948 | 0.032319 | ** p<0.01 |

**Bonferroni and Holm multiple comparison**

| **Treatments pair** | **Bonferroni and Holm T-statistic** | **Bonferroni**  **p-value** | **Bonferroni inference** | **Holm p-value** | **Holm inference** |
| --- | --- | --- | --- | --- | --- |
| Normal vs pH 4 | 2.3012 | 0.4416415 | ns | 0.1324924 | ns |
| Normal vs pH 6.5 | 1.2572 | 2.3723281 | ns | 0.4744656 | ns |
| Normal vs pH 9 | 18.3634 | 4.9361E-08 | ** p<0.01 | 0.00049361 | ** p<0.01 |
| Normal vs pH 11 | 4.5214 | 0.0110589 | * p<0.05 | 0.0044236 | ** p<0.01 |
| pH 4 vs pH 6.5 | 1.1312 | 2.8436219 | ns | 0.2843622 | ns |
| pH 4 vs pH 9 | 17.547 | 7.6812E-08 | ** p<0.01 | 0.0006145 | ** p<0.01 |
| pH 4 vs pH 11 | 5.966 | 0.0013824 | ** p<0.01 | 0.0008295 | ** p<0.01 |
| pH 6.5 vs pH 9 | 18.3534 | 4.9623E-08 | ** p<0.01 | 0.00044661 | ** p<0.01 |
| pH 6.5 vs pH 11 | 5.4055 | 0.0029909 | ** p<0.01 | 0.0014955 | ** p<0.01 |
| pH 9 vs pH 11 | 12.948 | 1.4247E-06 | ** p<0.01 | 0.0099727 | ** p<0.01 |

ns: not significant

**ANOVA: SINGLE FACTOR for nitrogen sources**

**Summary**

| **Groups** | **Count** | **Sum** | **Average** | **Variance** |
| --- | --- | --- | --- | --- |
| Normal | 4 | 10.26 | 2.57 | 0.0157 |
| BG11_0_ | 3 | 17.50 | 5.83 | 0.8436 |
| NH_4_Cl | 2 | 6.62 | 3.31 | 0.2326 |
| Urea | 3 | 7.59 | 2.53 | 0.0822 |

**ANOVA**

| **Source of Variation** | **SS** | **df** | **MS** | **F** | **P-value** |
| --- | --- | --- | --- | --- | --- |
| Between Groups | 22.71 | 3 | 7.57 | 28.41 | 0.0001 |
| Within Groups | 2.13 | 8 | 0.271 |  |  |
| Total | 24.84 | 11 |  |  |  |

H0:	Normal=BG110=NH4Cl=Urea

If p<0.05, we reject the null hypothesis, meaning at least one condition is different.

The p-value corresponding to the F-statistic of one-way ANOVA is lower than 0.05, **suggesting that one or more treatments are significantly different**. The Tukey HSD test, Scheffé, Bonferroni and Holm multiple comparison tests follow. These post-hoc tests would likely identify which of the pairs of treatments are significantly different from each other.

**Tukey HSD Test**

| **Treatments pair** | **Tukey HSD Q statistic** | **Tukey HSD p-value** | **Tukey HSD inference** |
| --- | --- | --- | --- |
| Normal vs BG11_0_ | 11.7179 | 0.0010053 | ** p<0.01 |
| Normal vs NH_4_Cl | 2.3605 | 0.3982094 | ns |
| Normal vs Urea | 0.1307 | 0.8999947 | ns |
| BG11_0_ vs NH_4_Cl | 7.5645 | 0.0030480 | ** p<0.01 |
| BG11_0_ vs Urea | 11.0833 | 0.0010053 | ** p<0.01 |

**Scheffé multiple comparison**

| **Treatments pair** | **Scheffé T-statistic** | **Scheffé p-value** | **Scheffé inference** |
| --- | --- | --- | --- |
| Normal vs BG11_0_ | 8.2858 | 0.0002795 | ** p<0.01 |
| Normal vs NH_4_Cl | 1.6691 | 0.4700905 | ns |
| Normal vs Urea | 0.0924 | 0.9997719 | ns |
| BG11_0_ vs NH_4_Cl | 5.3489 | 0.0050958 | ** p<0.01 |
| BG11_0_ vs Urea | 7.8371 | 0.0004135 | ** p<0.01 |
| Normal vs BG11_0_ | 1.6608 | 0.4740027 | ns |

**Bonferroni and Holm multiple comparison**

| **Treatments pair** | **Bonferroni and Holm**  **T-statistic** | **Bonferroni**  **p-value** | **Bonferroni inference** | **Holm p-value** | **Holm inference** |
| --- | --- | --- | --- | --- | --- |
| Normal vs BG11_0_ | 8.2858 | 0.0002033 | ** p<0.01 | 0.0002033 | ** p<0.01 |
| Normal vs NH_4_Cl | 1.6691 | 0.8018391 | ns | 0.4009195 | ns |
| Normal vs Urea | 0.0924 | 5.5718236 | ns | 0.9286373 | ns |
| BG11_0_ vs NH_4_Cl | 5.3489 | 0.0041206 | ** p<0.01 | 0.0027471 | ** p<0.01 |
| BG11_0_ vs Urea | 7.8371 | 0.0003037 | ** p<0.01 | 0.0002531 | ** p<0.01 |
| Normal vs BG11_0_ | 1.6608 | 0.8119740 | ns | 0.2706580 | ns |

ns: not significant

**ANOVA: SINGLE FACTOR for Urea**

**Summary**

| **Groups** | **Count** | **Sum** | **Average** | **Variance** |
| --- | --- | --- | --- | --- |
| Normal | 4 | 10.268 | 2.57 | 0.0157 |
| Urea 8 mM | 3 | 10.84 | 3.61 | 0.0808 |
| Urea 16 mM | 3 | 25.96 | 8.65 | 7.7515 |

**ANOVA**

| **Source of Variation** | **SS** | **Df** | **MS** | **F** | **P-value** |
| --- | --- | --- | --- | --- | --- |
| Between Groups | 68.67 | 2 | 34.3374 | 15.29 | 0.0029 |
| Within Groups | 15.71 | 7 | 2.2445 |  |  |
| Total | 84.39 | 9 |  |  |  |

H0:	Normal=8mM=16mM (Urea)

If p<0.05, we reject the null hypothesis, meaning **at least one condition is different.**

The p-value corresponding to the F-statistic of one-way ANOVA is lower than 0.05, **suggesting that one or more treatments are significantly different**. The Tukey HSD test, Scheffé, Bonferroni and Holm multiple comparison tests follow. These post-hoc tests would likely identify which of the pairs of treatments are significantly different from each other.

**Tukey HSD Test**

| **Treatments pair** | **Tukey HSD Q statistic** | **Tukey HSD p-value** | **Tukey HSD inference** |
| --- | --- | --- | --- |
| Normal vs 8mM | 12.9280 | 0.6430275 | insignificant |
| Normal vs 16mM | 7.5246 | 0.0027112 | ** p<0.01 |
| 8mM vs 16mM | 5.8293 | 0.0107466 | * p<0.05 |

**Scheffé multiple comparison**

| **Treatments pair** | **Scheffé T-statistic** | **Scheffé p-value** | **Scheffé inference** |
| --- | --- | --- | --- |
| Normal vs 8mM | 0.9142 | 0.673856 | insignificant |
| Normal vs 16mM | 5.3207 | 0.0034689 | ** p<0.01 |
| 8mM vs 16mM | 4.1220 | 0.0134186 | * p<0.05 |

**Bonferroni and Holm multiple comparison**

| **Treatments pair** | **Bonferroni and Holm**  **T-statistic** | **Bonferroni**  **p-value** | **Bonferroni inference** | **Holm p-value** | **Holm inference** |
| --- | --- | --- | --- | --- | --- |
| Normal vs 8mM | 0.9142 | 1.1730751 | insignificant | 0.3910250 | insignificant |
| Normal vs 16mM | 5.3207 | 0.0032952 | ** p<0.01 | 0.0032952 | ** p<0.01 |
| 8mM vs 16mM | 4.1220 | 0.0133460 | * p<0.05 | 0.0088973 | ** p<0.01 |

ns: not significant

**B2.** One-way ANOVA for *Coelastrella thermophila* D14 biomass grown in BG11 and different percentages of piggery wastewater (5, 10, and 20%) – culture and extract after high-pressure homogenization at 1200 bar and 1 cycle – as biostimulant: statistically significant results at ɑ = 0.05.

| **Concentration (g/L)** | **Tukey's multiple**  **comparisons test** | **Summary** | **Adjusted P Value** |
| --- | --- | --- | --- |
| 0.1 | BG11:Culture vs. 10%:Culture | ** | 0.0099 |
|  | BG11:Extract vs. 10%:Culture | * | 0.0301 |
|  | 5%:Extract vs. 10%:Culture | ** | 0.0027 |
|  | 10%:Culture vs. 10%:Extract | * | 0.0286 |
|  | 10%:Culture vs. 20%:Culture | ** | 0.0016 |
| 0.5 | 5%:Culture vs. 20%:Culture | *** | 0.0008 |
| 1 | BG11:Culture vs. BG11:Extract | * | 0.0147 |
|  | BG11:Culture vs. 5%:Extract | * | 0.0368 |
|  | BG11:Extract vs. 5%:Culture | *** | 0.0009 |
|  | 5%:Culture vs. 5%:Extract | ** | 0.0033 |
| 2 | BG11:Culture vs. BG11:Extract | **** | <0.0001 |
|  | BG11:Culture vs. 5%:Extract | ** | 0.0056 |
|  | BG11:Culture vs. 10%:Culture | **** | <0.0001 |
|  | BG11:Culture vs. 20%:Culture | * | 0.0113 |
|  | BG11:Culture vs. 20%:Extract | ** | 0.0021 |
|  | BG11:Extract vs. 5%:Culture | * | 0.0108 |
|  | BG11:Extract vs. 10%:Extract | ** | 0.0051 |
|  | 10%:Culture vs. 10%:Extract | * | 0.042 |
