# Supplementary material for: A solar panel-origin microalga, Coelastrella thermophila D14, with high potential for wastewater biotechnology
Source: Appl Microbiol Biotechnol. 2025 Nov 24;109(1):246. doi: 10.1007/s00253-025-13618-8 (PMC12647267; doi:10.1007/s00253-025-13618-8)
Supplement: Supplementary file 1 — (DOCX 833 KB) [file 253_2025_13618_MOESM1_ESM.docx]

**Supplementary Material A. Supplementary Tables and Figures**

**Table S1.** Assembly statistics for the *Coelastrella thermophila* D14 draft genome.

| **Parameters** | **Assembly statistics** |
| --- | --- |
| **Total length** | ~83 Mb |
| **Number of scaffolds / contigs** | 34,735 / 63,247 |
| **Number of scaffolds > 1 Kb** | 17,089 |
| **Number of scaffolds > 5 Kb** | 4,138 |
| **Number of scaffolds > 50Kb** | 52 |
| **Number of scaffolds > 100 Kb** | 8 |
| **Scaffold N50** | 3,568 (~5.6 Kb) |
| **Longest scaffold length** | ~195 Kb |
| **Gaps %** | 3.034 |
| **GC %** | 51.85 |

**Table S2.** Growth parameters of *Coelastrella* *thermophila* D14 growing in different conditions

| **Condition** | | **Doubling time^1^** | **Lag Phase^2^** | **OD_750nm_ Max^3^** |  |
| --- | --- | --- | --- | --- | --- |
| Control | | 2.57 ± 0.11 | 2 | 4.94 ± 0.70 |  |
| NaCl | 0.1 M | 1.99 ± 0.09 | 2 | 5.64 ± 0.36 |  |
|  | 0.25 M | 1.54 ± 0.08 | 2 | 5.13 ± 0.59 |  |
|  | 0.5 M | 1.60 ± 0.08 | 5 | 3.69 ± 0.31 |  |
|  | 1 M | nd | nd | nd |  |
| pH | 4 | 2.09 ± 0.12 | 5 | 2.22 ± 0.33 |  |
|  | 6.5 | 2.34 ± 0.07 | 3 | 3.69 ± 0.15 |  |
|  | 9 | 5.91 ± 0.26 | 3 | 1.67 ± 0.06 |  |
|  | 11 | 3.39 ± 0.30 | 3 | 1.93 ± 0.11 |  |
| Nitrogen sources | BG11_0_ | 5.83 ± 0.75 | 4 | 0.30 ± 0.04 |  |
|  | NH_4_Cl | 3.31 ± 0.34 | 2 | 1.02 ± 0.04 |  |
|  | Urea | 2.53 ± 0.23 | 4 | 2.63 ± 0.10 |  |
| Urea | 8 mM | 3.61 ± 0.23 | 2 | 4.01 ± 0.34 |  |
|  | 16 mM | 8.65 ± 2.27 | 4 | 2.60 ± 0.14 |  |
| ^1^Mean of doubling time in days ± the standard deviation (n=3). ^2^Lag phase expressed in days. ^3^OD_750nm_ maximal after 10 days of growth. nd: not determined (no growth). Conditions correspond to Fig. 3, Fig. 4A,B and Fig. 5A,B, respectively. | | | | | |

**Table S3.** Antibiotic sensitivity of *Agrobacterium* *tumefaciens* and *Coelastrella thermophila* D14.

| **Strain** | **Cefotaxime sodium salt (mg L^-1^)** | **Hygromycin (mg L^-1^)** | | |
| --- | --- | --- | --- | --- |
|  | 500 | 10 | 50 | 20 |
| *C. thermophila* D14 | + | - | - | - |
| *A. tumefaciens* LBA4404 + pCAMBIA1301 | - | + | + | + |

+: growth, -: no growth. Two biological replicates were made for every assay.

**
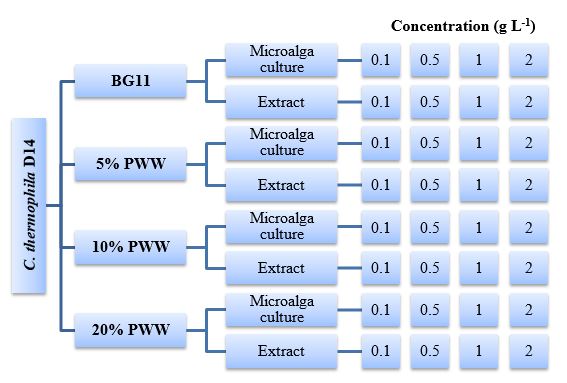
**

**Fig. S1.** Schematic diagram of the treatments of *Coelastrella thermophila* D14 tested in the germination trials. Different growth media (BG11, 5, 10, and 20% PWW), different biomass processing (microalga culture and extract from disrupted biomass after high-pressure homogenization at 1200 bar for 1 cycle), and different treatment concentrations (0.1, 0.5, 1, and 2 g L^-1^) were tested.


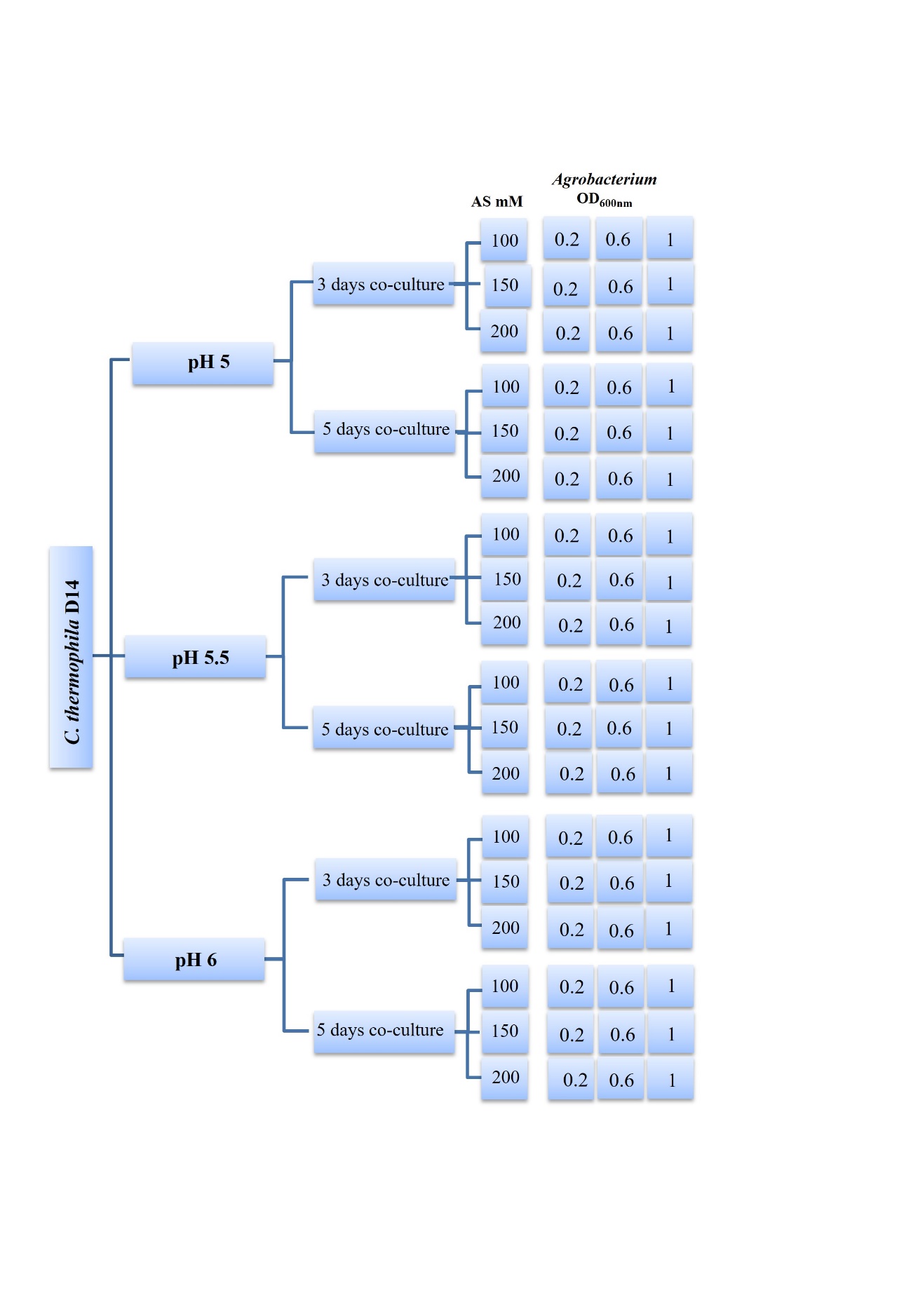


**Fig. S2.** All combinations tested in *Coelastrella thermophila* D14 transformation with *Agrobacterium tumefaciens* LB4404 + pCAMBIA1301. AS: acetosyringone

**
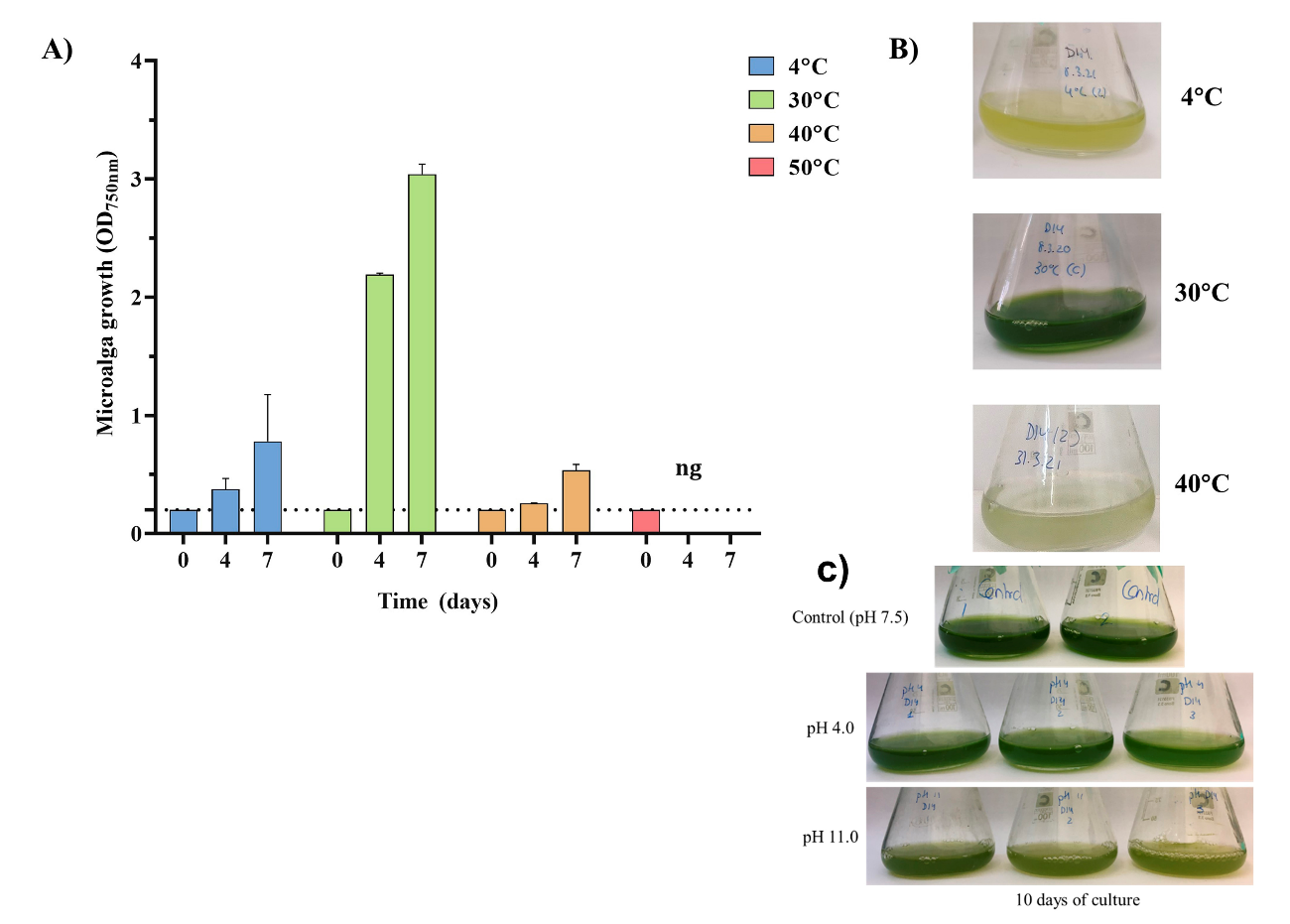
**

**Fig. S3.** Growth of *Coelastrella* *thermophila* D14 at different temperatures and pH. **A)** OD_750nm_ reached after 10 days of growth at 4, 30, 40, and 50 °C. The dashed line represents the initial optical density at 750 nm (OD_750nm_) of 0.2. **B)** Pictures taken from different points of the growth at 4, 30, and 40°C. At 50°C, there was no growth (ng). **C)** Pictures taken of the *C. thermophila* D14 cultures after 10 days of growth at pH 7.5 (control), 4.0, and 11.0.
